# Reviewing digital collaborative interactions with multimodal hyperscanning through an ever-growing database

**DOI:** 10.3389/fnrgo.2026.1756956

**Published:** 2026-02-10

**Authors:** Anna Vorreuther, Anne-Marie Brouwer, Mathias Vukelić

**Affiliations:** 1 Applied Neurocognitive Systems, Institute of Human Factors and Technology Management IAT, University of Stuttgart, Stuttgart, Germany; 2 Artificial Intelligence, Donders Centre for Brain, Cognition and Behavior, Radboud University, Nijmegen, Netherlands; 3 Human Performance, Netherlands Organization for Applied Scientific Research (TNO), Soesterberg, Netherlands; 4 Applied Neurocognitive Systems, Fraunhofer-Institute for Industrial Engineering IAO, Stuttgart, Germany

**Keywords:** collaboration, database, digitalization, electroencephalography, eye-tracking, functional near-infrared spectroscopy, hyperscanning, review

## Abstract

**Introduction:**

Digital technologies now mediate a substantial proportion of human collaboration, reshaping how individuals coordinate attention, share information, and jointly act on goals. These digitally mediated interactions engage neural, physiological, and behavioral processes differently compared to face-to-face settings. Mobile hyperscanning, i.e., simultaneous (neuro-)physiological measures of two or more individuals, offers a unique window into these multidimensional dynamics. Yet, the existing literature is highly fragmented in design, modality, and analytic rigor, making it difficult to accumulate knowledge. This review systematically synthesizes hyperscanning research investigating collaboration involving digital components and identifies key methodological and conceptual gaps that must be addressed to advance the field.

**Methods:**

We searched Scopus, PubMed, and Web of Science (April 2025) for mobile hyperscanning studies on digital collaboration. Forty-five eligible studies involving simultaneous measurements of at least two healthy adults engaged in collaborative tasks with a digital interaction component were included. Studies were categorized across 13 dimensions, including modality, task design, interaction type, analysis method, and cognitive domain. To ensure transparency and support cumulative synthesis, we created a continuously updated online resource (“InterBrainDB”).

**Results:**

Most studies relied on unimodal neuroimaging, predominantly electroencephalography (EEG) or functional near-infrared spectroscopy (fNIRS), with only seven studies implementing multimodal combinations. Study designs favored cooperative tasks or naturalistic scenarios with symmetrical roles, typically using same-sex dyads of unfamiliar individuals. Non-verbal interaction was studied slightly more often than verbal. Analytically, functional connectivity dominated, whereas effective connectivity, multimodal fusion, and machine learning were scarcely used. Executive and social cognition were more frequently investigated than creativity, memory, and language.

**Discussion:**

Research on digital collaboration through hyperscanning is growing, yet progress is limited by methodological heterogeneity, narrow use of modalities, and analytical conservatism. Future advances will require: (1) multimodal integration to fully capture neural, physiological, and behavioral dynamics; (2) systematic comparisons across varying degrees of digitalization to understand how technology shapes interaction; (3) physiology-informed analysis frameworks capable of modeling high-dimensional interpersonal dynamics; and (4) clearer reporting standards to enable reproducibility and large-scale synthesis. Resources like our InterBrainDB can structure a community-driven progress toward ecologically grounded models of digitally mediated collaboration, a domain of increasing scientific and societal relevance.

## 1 Introduction

### 1.1 Measuring collaboration in the digital age

Collaboration, i.e., an activity where two or more individuals share a goal or intention that motivates joint work (Léné et al., 2021; Wood and Gray, 1991), is one of the most prevalent topics under study in hyperscanning research. Much of the field has examined face-to-face social interaction under naturalistic conditions (Czeszumski et al., 2020; Fan et al., 2021; Réveillé et al., 2024; Schneider et al., 2021). Yet, collaboration in contemporary society increasingly occurs through digital means, including screen-based meetings, shared online workspaces, and immersive virtual environments (Deschênes, 2024; Wu et al., 2023; Yang et al., 2025). These developments raise critical questions about how environmental conditions (virtual vs. in-person) and task-specific tools or modalities (e.g., digital vs. analog objects, verbal vs. text-based communication) influence the brain, body, and behavioral signatures of collaboration (Leahy et al., 2025; Magni et al., 2025; Solomon and Theiss, 2022). Digital media introduces a virtual divide (e.g., a monitor), altering attentional demands (Chuang and Hsu, 2023; Snijdewint and Scheepers, 2023), perception of implicit social cues (Frith and Frith, 2008; Oh et al., 2018; Sharan et al., 2022), and cognitive workload (Luebstorf et al., 2023; Nurmi and Pakarinen, 2023). As a result, findings from face-to-face hyperscanning studies cannot be assumed to generalize to digitally mediated settings, where collaborators rely on virtual rather than physical co-presence (Balconi et al., 2022; Balters et al., 2023; Liu et al., 2019; Sarasso et al., 2022, 2024). Although prior work suggests that the degree of digitality modulates neural and behavioral responses (for reviews, see Balters et al., 2020; Barde et al., 2020), the literature remains fragmented by different choices in task, measurement, and analysis. These developments motivate a systematic review of how hyperscanning is currently used to investigate digital collaboration.

### 1.2 From hyperscanning origins to multimodal mobile neuroimaging

The term hyperscanning was introduced by (Montague et al. 2002) to describe simultaneous functional magnetic resonance imaging (fMRI) of interacting individuals playing a competitive deception task. Earlier work had already explored dual-brain recordings and reported synchronized electroencephalography (EEG) activity in the alpha band (8–13 Hz) in identical twins (Duane and Behrendt, 1965). Since then, hyperscanning has expanded rapidly, driven by advances in mobile neuroimaging (for a review on the historical development see Nam et al., 2020). Today, both EEG and functional near-infrared spectroscopy (fNIRS) are the most widely used mobile brain-imaging modalities, offering complementary strengths: EEG provides high temporal resolution on a millisecond timescale, whereas fNIRS affords greater spatial specificity for cortical surface activity, making it well-suited for mapping spatial patterns of brain activation (for review see Mehta and Parasuraman, 2013). Recent advances in mobile neuroimaging and physiological sensing, together with increasingly accessible wearable sensors, have expanded hyperscanning beyond stationary setups such as fMRI or magnetoencephalography (MEG), enabling the study of interpersonal dynamics in increasingly diverse and applied settings (Carollo and Esposito, 2024).

Real-world and digitally mediated interactions unfold across multiple sensory channels and involve tightly coupled cognitive, affective, and behavioral processes (Zamm et al., 2024). Accordingly, reviews increasingly emphasize the value of multimodal and ecologically grounded frameworks of social interaction (Hakim et al., 2023; Schneider et al., 2021). Empirical work has begun to integrate brain-based methods with bodily measures such as gaze alignment (Chuang and Hsu, 2023), electrocardiography (ECG), electrodermal activity (EDA; Numata et al., 2021), communication signals (Lu et al., 2020), and other camera-based physiological metrics (Shih et al., 2024). This integration, called embodied hyperscanning (for review see Grasso-Cladera et al., 2024), has facilitated the transition from controlled laboratory paradigms to applied settings such as classrooms (Dikker et al., 2017; Zhang et al., 2024), workplaces (Wikström et al., 2021; Wu et al., 2023), and other real-world environments (Balters et al., 2023). In line with this evolution, we use the term “hyperscanning” here to encompass approaches that incorporate at least one brain- or body-based measurement modality of physiology.

### 1.3 Established and emerging analytic approaches

Irrespective of the signal measured, inter-brain synchrony (IBS) has been the most widely reported analysis approach for interpersonal dynamics in a variety of interactive scenarios (for review see Réveillé et al., 2024). IBS has been linked to various processes central to social interaction, including shared attention (Dikker et al., 2017; Snijdewint and Scheepers, 2023), simultaneous movements (Gumilar et al., 2021), imitation (Delaherche et al., 2015; Konvalinka et al., 2023), social closeness between partners (Reinero et al., 2021), and the nature of their engagement (e.g., cooperation vs. competition; Hayne et al., 2023). Given the pronounced sensitivity of IBS to contextual and situational factors, newly emerging digital collaboration formats warrant systematic investigation as they increasingly reflect naturalistic settings (Fan et al., 2021). Alongside synchrony-based metrics on the inter-brain level, multivariate modeling and machine learning have emerged in intra-brain research that could expand the analytical repertoire for hyperscanning, enabling improved decoding of interpersonal states and complementary use of EEG, fNIRS, and physiological signals (for reviews see Dissanayake et al., 2025; Lühmann and Müller, 2017; Pinto-Orellana et al., 2024).

### 1.4 Challenges and structuring principles in the literature

Despite these methodological and analytic advances, synthesizing hyperscanning studies investigating digital social interaction remains challenging due to substantial heterogeneity. Heterogeneity is introduced by varying group size (dyads, triads, or larger collectives; Hou et al., 2022; Park et al., 2023), pre-existing social relationships between partners (Bae et al., 2024; Dikker et al., 2021), and the gender composition of paired participants (Zhang et al., 2023c). Furthermore, the degree of physical vs. virtual co-presence differs across paradigms with respect to how participants are situated (same or different rooms, physical or virtual co-presence), how information is exchanged, and to what extent interactions rely on digital vs. analog means (Balters et al., 2020). For instance, some experiments manipulate remoteness by prohibiting direct visual and/or auditory contact through a physical divide (e.g., Wu et al., 2023) or by placing participants back-to-back (e.g., Kütt et al., 2019).

Previous reviews have proposed frameworks to organize this diversity. For digitally mediated interaction, several categories have been identified by (Balters et al. 2021): (i) the type of communication (goal-directed vs. open-ended), (ii) the transfer of information (i.e., whether information exchange between participants happened via analog, digital, or mixed channels), (iii) the interaction medium (originally referred to as “interaction manipulative”) being digital or non-digital, and (iv) the interaction scenario, i.e., the spatial layout of how participants are situated relative to each other (e.g., face-to-face, side-by-side, virtually connected). Other work identified the experimental task itself as a central factor for recurring experimental archetypes (Barde et al., 2020; Wang et al., 2018). Moreover, a clear conceptual distinction can be made between collaborative interactions based on task symmetry, distinguishing between symmetrical interactions without predefined roles and asymmetrical tasks with designated roles (Wikström, 2022). Together, these dimensions outline the core design space of hyperscanning studies, albeit they are currently not integrated across modalities or applied specifically to digitally mediated collaboration.

### 1.5 Objective of the present review

Without structuring the body of research considering all these dimensions, theoretical synthesis and cross-study comparison remain difficult, particularly when assessing how digitalization shapes the neural, physiological, and behavioral foundations of collaboration. The current review organizes hyperscanning research along dimensions relevant to studying remote and digitally mediated collaboration. Building on this structured synthesis, we present the framework for a living literature database: an extensible, interactive platform that categorizes hyperscanning studies according to measurement modalities, analytic approaches, interaction design dimensions, and task characteristics. The current version includes studies of remote collaborative interactions using mobile hyperscanning methods, with the long-term goal of expanding the database to incorporate additional modalities, paradigms, and newly published work.

## 2 Methods

**Figure 1:**
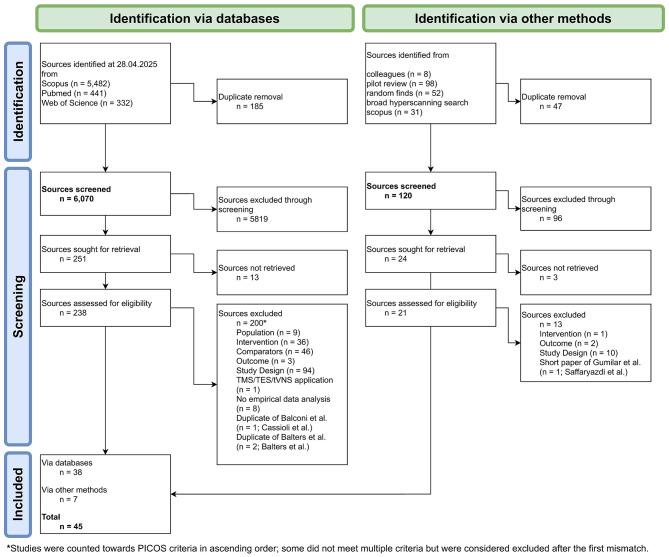
Overview of source retrieval. Flowchart of source retrieval process according to PRISMA guidelines.

This review followed the Preferred Reporting Items for Systematic Reviews and Meta-Analyses (PRISMA) guidelines (Liberati et al., 2009; Page et al., 2021). A summary of the retrieval, screening, and inclusion steps can be found in Figure 1.

### 2.1 Identification

To identify eligible sources for review, a broadly defined search for hyperscanning studies, including or focusing on digital interaction scenarios, was conducted using a combination of search terms that reflect hyperscanning, collaboration, recording modalities, and digital context (see Table 1). Initially, Scopus (https://www.scopus.com), PubMed (https://pubmed.ncbi.nlm.nih.gov), and Web of Science (https://www.webofscience.com) were searched. This search was conducted on April 28, 2025. The exact search terms were adapted according to the specifications of each search engine (for details, see Supplementary Table 1). The initial search was carried out for journal articles published in English or German, searching paper titles and abstracts only. In line with the PRISMA guidelines, additional (unsystematic) data searches were conducted as well (see Figure 1). Any additional findings brought to us via search alerts or by colleagues were included if eligible, although no additional proactive search was conducted.

**Table 1:** Concepts contained in search string components.

**Concept**	**Search string component**
Hyperscanning	(hyperscan^*^ OR “social neuroscience” OR “two-person neuroscience” OR interbrain OR interpersonal OR “brain-to-brain interaction” OR interneural OR inter-subject OR synchron^*^ OR coupling OR “functional connectivity” OR “effective connectivity”)
Collaboration	(team OR “team performance” OR “team engagement” OR “team dynamics” OR “group performance” OR “group dynamics” OR “collaborative engagement” OR “collaborative performance” OR “collective efficacy” OR flow OR “team flow” OR “work flow” OR “group flow” OR “collective flow”)
Recording modalities	(fnirs OR “functional near-infrared spectroscopy” OR eeg OR electroencephalogra^*^ OR ecg OR electrocardiogra^*^ OR ppg OR photoplethysmogra^*^ OR eda OR “electrodermal activity” OR “heart rate” OR pulse OR “skin conductance” OR eye-track^*^ OR “eye tracking” OR “gaze tracking” OR physiolog^*^ OR multimodal^*^ OR “multi-modal”)
Digital context	(remote OR virtual OR online OR web-based)

Search string components reflect the four conceptual domains used in the systematic database search: hyperscanning terminology, collaboration-related constructs, mobile neurophysiological and physiological recording modalities, and digital or remote interaction contexts. Quotation marks indicate exact phrase matching; asterisks denote wildcard operators.

Following the PRISMA recommendation, we used the PICOS eligibility criteria (Population, Intervention, Comparison, Outcomes, Study design) to assess eligibility (see Table 2). For the presented review, we considered only studies with a *population* of at least two clinically healthy adult participants. As an eligible experimental *intervention*, we defined the application of one or more mobile brain or body imaging techniques for physiological data to focus on mobile and applied neuroergonomics contexts. We did not limit the search to one specific mobile method since we aimed to investigate the number of joint applications of measurements. Note that we also identified some studies using camera-based tracking, encompassing the assessment of eye contact and facial muscle movements. However, this modality was not explicitly included in the search scope. Nevertheless, a specific label was assigned in these cases, given that the modality might be of interest. For the *comparison* criterion, we included studies that scanned at least two participants simultaneously during an interaction. Given the search term, we expected to find mainly interaction scenarios with group-based collaboration. As *outcomes*, we defined any results addressing inter-subject dynamics as assessed by the measurement modalities of interest. Studies reporting no statistical results related to the physiological measures during the collaborative interactions were not included. Lastly, we included the additional *study design* criterion to limit the review to hyperscanning studies that introduced a collaborative scenario with a focus on a digital aspect in the study design. For example, studies meeting all initial criteria and focusing purely on face-to-face collaboration were excluded. Further, studies with tasks involving imitation rather than autonomous choice of all collaborators were not included (Bieńkiewicz et al., 2021).

**Table 2:** PICOS eligibility criteria applied during source selection.

**PICOS criterion**	**Description**
Population	Two or more clinically healthy adult participants
Intervention	fNIRS, EEG, EDA, ECG, PPG, and eye-tracking are applied to acquire data for analysis
Comparison	Scanning two or more participants simultaneously during interaction
Outcomes	Inter-subject dynamics analysis
Study design	Collaborative scenario with one or more digital interaction media or scenarios

ECG, Electrocardiography; EDA, Electrodermal Activity; EEG, Electroencephalography; fNIRS, Functional Near-Infrared Spectroscopy; PPG, Photoplethysmography.

### 2.2 Duplicate removal

For duplicate removal, the Zotero (version 6.0.36) and Citavi (version 6.15) software were used, as well as an automated matching of cosine similarity of titles and abstracts using sklearn (Pedregosa et al., 2011). Finally, the web-based review program Rayyan (Ouzzani et al., 2016) was used for duplicate removal and further screening procedures. All automated duplicate removals were manually confirmed before exclusion.

### 2.3 Screening

The identification procedure followed by duplicate removal resulted in 6,070 sources from the database searches and 120 sources from other methods (see Figure 1).

#### 2.3.1 Initial screening

We adopted a liberal inclusion policy for every step of the screening process, i.e., we opted to include a source one step further during screening rather than excluding a source pre-emptively. Given the large amount of retrieved literature, we first screened titles to exclude some search results that were unlikely to meet the inclusion criteria. Titles including words or phrases like “rodents,” “non-human,” “epilepsy,” or “Alzheimer” were excluded, assuming that the Population criterion would not be met. Indications of the use of transcranial magnetic stimulation, transcranial electrical stimulation, or transcutaneous vagus nerve stimulation were another exclusion reason. Furthermore, several titles indicated that the source had no relation to the field of neuroscience in general and instead related to the physics of water flow or aerodynamics. Hereafter, we screened sources for eligibility based on both title and abstract.

#### 2.3.2 Retrieval and manuscript screening for eligibility

After the initial title and abstract screening, the full-text versions of sources were retrieved. If full-text versions were not immediately retrievable (e.g., through open-source access), an effort was made to obtain the full-text version by means of university access rights or by contacting the corresponding author once via email to request access. We successfully retrieved 238 sources derived from the systematic search and an additional 21 from other methods. To screen and label sources for eligibility for the current review, the Rayyan software was used (Ouzzani et al., 2016). For each review stage, sources were included rather than excluded to decrease the chance of false exclusion. The eligibility criteria would be met with certainty in the final full-text review stage. Screening of the full manuscripts led to the exclusion of an additional 213 sources (for details, see Figure 1). In summary, a total of 45 sources were included in this review. Note that, given that the criteria for *study design* and *comparison* often required in-depth reading of the methods and results sections to ensure thorough screening, many unsuitable sources were initially included in the full-text stage. Hence, those criteria were the most common late-stage exclusion reason (see Figure 1).

### 2.4 Extraction of information and categorization

After collecting relevant sources based on the PRISMA criteria, we aimed to extract commonalities across studies by categorizing studies across 13 dimensions based on design choices, measurement modalities, analysis approaches, and targeted cognitive functions. Category definitions were adapted from existing hyperscanning reviews (e.g., Balters et al., 2020, 2021; Wang et al., 2018) or based on influential factors of the sample and design (e.g., how auditory and visual information is exchanged between participants during the interaction). Each included source was individually reviewed and subsequently categorized by extracting the following information: (1) measurement modalities of interest used in the study (e.g., EEG, eye-tracking); (2) the number of participants included in analyses; (3) the pairing configuration of participants (whether measurements were taken simultaneously on dyads, triads, tetrads, or larger groups; Hou et al., 2022; Park et al., 2023); the pairing setup of participants, specifically variables of the setup that are known to influence behavior and subsequently results, such as (4) gender (Zhang et al., 2023c) and (5) relationship (Bae et al., 2024; Dikker et al., 2021) of simultaneously measured individuals; (6) the type of hyperscanning paradigm employed (see Table 3; Barde et al., 2020; Wang et al., 2018); (7) the task symmetry (Wikström, 2022), where highly symmetrical tasks allow participants to assume equal roles (e.g., puzzling with shared pieces), whereas low-symmetry tasks involve distinct roles (e.g., navigator and pilot); (8) the type of communication defined as either open-ended or goal-driven, and (9) the transfer of information being either analog, digital, or mixed (Balters et al., 2020). Following the detailed interaction categories introduced by (Balters et al. 2021) for digital hyperscanning studies, the (10) interaction scenario during the experiment was assessed (i.e., how participants were situated in relation to each other during measurements) and (11) whether the experiment included verbal, physical, and/or digital interaction media in shared or separated manners (for details see Table 4; Balters et al., 2021). Given the diverse measurement modalities included in the present review, we aimed to categorize (12) analysis methods in a signal-agnostic manner, i.e., not tied to specific modalities like EEG or fNIRS (Hakim et al., 2023). Therefore, we distinguished on a higher level between the analysis approaches focused on temporal aspects, spatial distribution, and connectivity domains, as well as machine learning methods (for details, see Table 5). Finally, we attempted to highlight the (13) cognitive function of interest investigated primarily during analyses for each study (Balters et al., 2021). Note that multiple labels within one category may apply to the same study.

**Table 3:** Paradigm category descriptions.

**Paradigm category**	**Description**
Cooperation and competition tasks	Participants either collaborate toward a shared goal or compete against one another
Coordination tasks	Participants perform a task that requires actions to be coordinated in time across partners
Ecologically valid scenarios	Participants are placed in real-world interaction contexts while under neuroimaging
Economic exchange tasks	Participants exchange a type of currency (either real or artificially constructed for the experiment)
Eye contact/gaze-based tasks	Participants look at each other and/or follow the gaze of another
Imitation tasks	Participants imitate the other's movement or behavior

The paradigm categories were originally derived by (Wang et al. 2018). Note that imitation and economic exchange tasks were included to provide a complete overview of categories, although none of the included studies in this review fall within those categories.

**Table 4:** Categories of interaction media.

**Interaction medium**	**Description**
Shared physical IM and verbal IM	Participants share physical objects and verbally communicate while interacting with the objects
Physical IM w/out verbal IM	Participants interact with non-shared tangible interfaces or musical instruments without verbal communication
Verbal IM	Participants solely interact verbally without any physical or non-verbal communication
Non-verbal IM	Participants solely interact non-verbally, such as looking at one another or synchronizing limb movements while observing one another
Shared digital IM and verbal IM	Participants interact together on one shared computer screen while also engaging in verbal interaction
Separate digital IM and verbal IM	Participants interact together on separate computer screens while also engaging in verbal interaction
Shared digital IM w/out verbal IM	Participants interact together on one shared computer screen without verbally communicating.
Separate digital IM w/out verbal IM	Participants interact on separate digital task media without interacting verbally

The interaction medium categories were derived from (Balters et al. 2021), who defined those to cluster fNIRS hyperscanning research involving digital components. Note that the category was originally termed “interaction manipulative.”

**Table 5:** Categories of multimodal analysis methods.

**Analysis domain**	**Approach**	**Description**
Connectivity	Functional	Quantifies undirected statistical associations (e.g., correlation, synchrony) between signals from two or more individuals. Applicable to any time-series data
	Effective	Assesses directed influence or causal relationships between signals across individuals (e.g., Granger causality, transfer entropy, or directional mutual information). Applicable to any time-series data
Temporal domain	Time-based	Analyzes time-aligned patterns in signals, capturing moment-to-moment fluctuations (e.g., alignment in gaze, HRV bursts, EEG amplitude). Applicable to signals with uniform time alignment
	Frequency-based	Decomposes signals into frequency components to analyze rhythmic coupling (e.g., alpha-band EEG, cardiac-respiratory oscillations). Applicable to oscillatory modalities
Spatial domain	Sensor-level	Uses data at the measurement site (e.g., EEG electrode, fNIRS optode), enabling cross-participant topographic comparisons or coupling at sensor locations. Applicable to spatially distributed signals
	Source-level	Projects sensor-level data into source space (e.g., cortical regions or gaze location on screen), allowing spatially informed inter-individual comparisons. Applicable to spatially distributed signals
Machine learning	Supervised	Mappings are learned from multimodal signal features to predefined labels (e.g., task success, social outcome) across dyads or groups, enabling prediction or classification of interaction dynamics. Applicable to any signal modality
	Unsupervised	Discovers latent patterns, clusters, or dimensions in joint participant data without labels (e.g., synchronized state clusters, engagement modes). Applicable to high-dimensional or multivariate data of any sensor modality
Other		Grouping label of any analysis method that does not match the above categories

The inclusion of various measurement modalities requires broad category definitions for analysis methods employed in studies.

#### 2.4.1 Living literature review database ≫InterBrainDB≪

**Figure 2:**
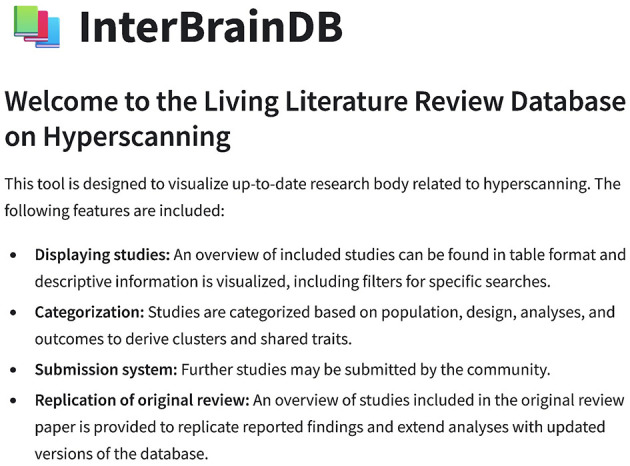
Screenshot of living literature review platform. A screenshot of the platform hosting the living literature review built using the Python library Streamlit (version 1.45.1).

Building on the initial review, a continuously updated “living” literature review was launched and maintained to track emerging research on multimodal hyperscanning (see Figure 2). The app utilizes the categories as described in the previous section and includes an interactive visualization tool, serving as a dynamic, open-access resource. To build the interactive platform the Python library *streamlit* (version 1.45.1; Streamlit Inc., 2025) was used, supporting the browsers Google Chrome, Firefox, Microsoft Edge, and Safari. The database allows filtering by sample population, interaction design, modality, analysis method, and cognitive domain, and will be expanded to incorporate additional modalities and future publications. Thus, the purpose of the database is two-fold: (1) it is intended to supplement the present review by allowing for replication of results and figures, as well as keeping the present analyses up-to-date by integrating newly published, relevant literature; (2) it is intended as a tool for the scientific community to search for literature based on the introduced categories and be extended to include hyperscanning studies with digital components beyond the presented PICOS criteria. To achieve this long-term value, sources submitted by the scientific community via the app's anonymous submission form are reviewed and categorized monthly by the first author continuously over time. Moreover, categorization may be proposed by the users upon submission of a source and changes to existing labels can be requested by contacting the first author. The server-hosted living review can be found here: https://websites.fraunhofer.de/interbraindb, with source code hosted at https://github.com/acv132/InterBrainDB. So far, 106 studies have been categorized and included in the online database. Studies in the database comprise the studies included in this review paper as indicated within the database itself. Furthermore, hyperscanning studies not strictly meeting the presented PICOS criteria were included and categorized, such as studies with underage sample populations, non-mobile imaging (e.g., fMRI), or paradigms beyond digital collaboration (e.g., face-to-face collaboration). When initially opening the app, the default filter configuration includes only sources reported in this paper, allowing for easy replication of results and interactive figures. By customizing filters in the sidebar, the database enables users to perform analyses according to their preferences and to download figures, reference tables, and BibTex files.

## 3 Results

### 3.1 Study selection

**Figure 3:**
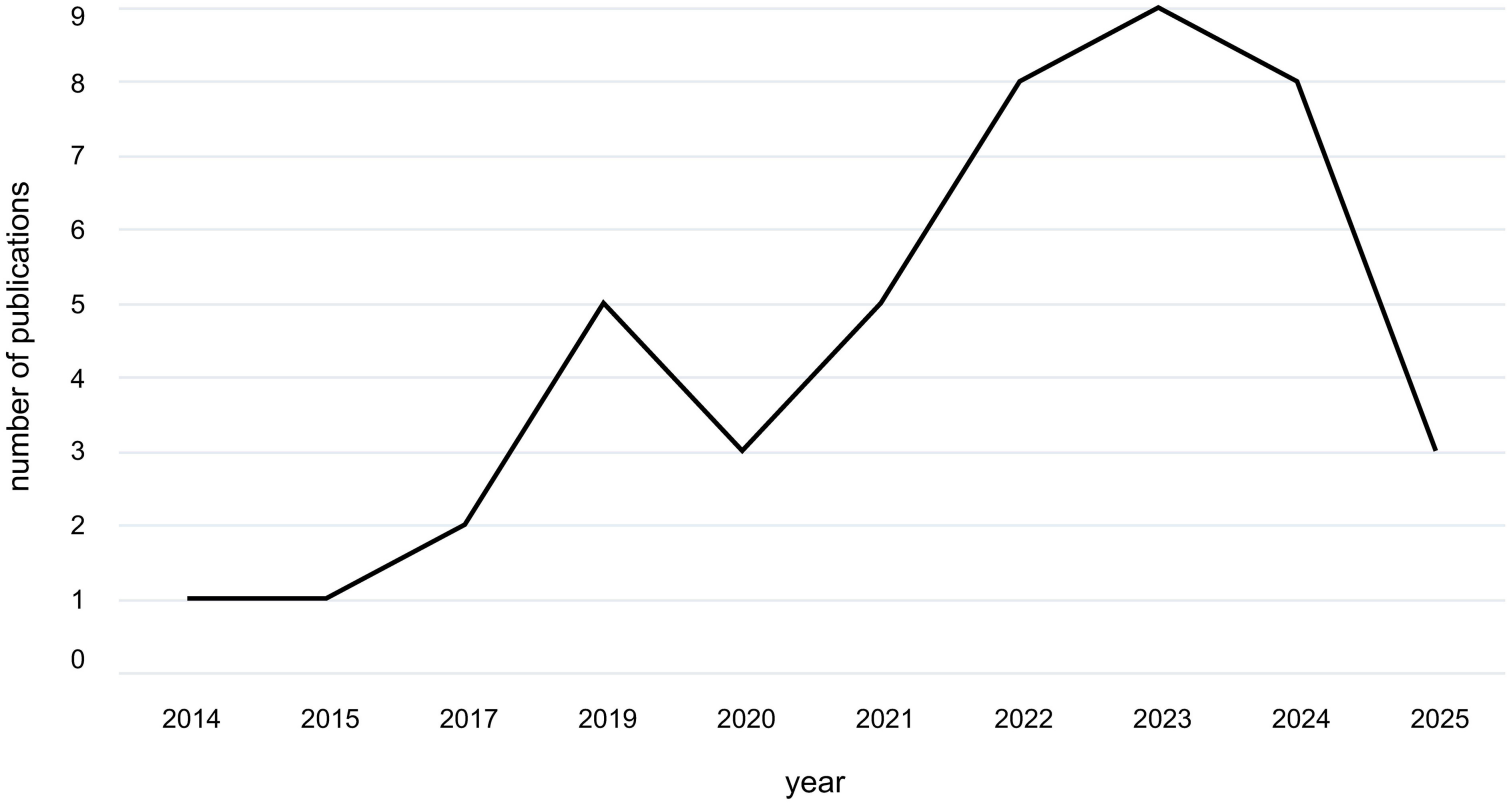
Number of publications by year. The number of publications selected for this review plotted over the years. The earliest included studies were published in the mid-2010s (Melendez-Calderon et al., 2015; Strang et al., 2014) with a steady increase in the number of publications following thereafter, peaking with *n* = 9 in 2023. Note a noticeable dip in published studies occurred following the onset of the COVID pandemic at the end of the year 2019.

An overview of the 45 included studies can be found in Table 6. The earliest studies included were published in the mid-2010s (see Figure 3). The average sample size across studies was 55.8 ± 70.3 (SEM = 10.48). The sample sizes varied considerably, from small-scale studies with four (Pöysä-Tarhonen et al., 2021) to large-scale studies with 480 participants (Zhang et al., 2023b). Most studies included within-subjects designs (*n* = 33), some analyzed between-subjects effects (*n* = 10), and two studies had a mixed design (Fang et al., 2022; Yamaya et al., 2025). This observed distribution might be attributed to the fact that sufficient statistical power of a between-subjects design requires a substantially larger recruitment effort for hyperscanning studies.

**Table 6:** Overview of studies.

**References**	**Measurement modalities**	**Participants; group size, gender of pairs [fm, ff, mm], familiarity**	**Paradigm**	**Task symmetry**	**Type of communication**	**Transfer of information**	**Interaction scenario**	**Interaction medium**	**Type of analysis**	**Cognitive function**
**Multimodal measurements**										
(Balconi et al. 2022)	[“ECG,” “EDA,” “EEG”]	*N* = 20; dyad, n/s, [“unfamiliar,” “instructor-student”]	Ecologically valid setting	Low	Open-ended	Mixed	[“FTF,” “virtual”]	[“Verbal IM,” “separate digital IM and verbal IM”]	[“Connectivity functional,” “temporal domain frequency,” “spatial domain source”]	Social cognition
(Chuang and Hsu 2023)	[“EEG,” “eye-tracking”]	*N* = 58; dyad, [“ff,” “fm,” “mm”], n/s	[“Eye-contact/gaze-based tasks,” “cooperation/competition tasks”]	High	Goal-driven	Digital	[“FTF v-b,” “virtual”]	Separate digital IM w/out verbal IM	[“Connectivity functional,” “temporal domain time,” “temporal domain frequency,” “spatial domain sensor”]	[“attention,” “visuospatial cognition”]
(Gugnowska et al. 2022)	[“EEG,” “camera-based tracking”]	*N* = 28; dyad, [“ff,” “fm,” “mm”], unfamiliar	Ecologically valid setting	Low	Open-ended	Digital	Virtual	Physical IM w/out verbal IM	[“Spatial domain sensor,” “connectivity functional,” “temporal domain frequency”]	[“motor,” “other”]
(Numata et al. 2021)	[“ECG,” “EDA,” “other”]	*N* = 9; dyad, mm, familiar	Ecologically valid setting	Low	Mixed	Digital	Virtual	Separate digital IM and verbal IM	Temporal domain time	[“memory,” “executive function”]
(Shih et al. 2024)	[“fNIRS,” “camera-based tracking”]	*N* = 6; dyad, [“ff,” “fm,” “mm”], familiar	Ecologically valid setting	High	Open-ended	Mixed	[“SBS,” “virtual”]	[“Separate digital IM and verbal IM,” “separate digital IM w/out verbal IM”]	[“Temporal domain time,” “connectivity functional,” “spatial domain sensor”]	[“other,” “executive function”]
(Snijdewint and Scheepers 2023)	[“ECG,” “other”]	*N* = 117; triad, [“ff,” “fm,” “mm”], [“familiar,” “unfamiliar”]	Cooperation/competition tasks	Low	Goal-driven	Digital	[“FTF,” “SBS v-b”]	Separate digital IM w/out verbal IM	[“Temporal domain time,” “connectivity functional”]	[“attention,” “executive function”]
(Wang et al. 2024)	[“EEG,” “eye-tracking”]	*N* = 10; dyad, n/s, n/s	Ecologically valid setting	High	Open-ended	Digital	Virtual	Separate digital IM w/out verbal IM	[“Temporal domain time,” “temporal domain frequency,” “connectivity functional”]	Executive function
**EEG**										
(Astolfi et al. 2020)	EEG	*N* = 32; dyad, mm, unfamiliar	Cooperation/competition tasks	High	Goal-driven	Digital	FTF v-b	Separate digital IM w/out verbal IM	[“Connectivity effective,” “temporal domain frequency,” “spatial domain sensor,” “machine learning supervised,” “other”]	Visuospatial cognition
(Balconi et al. 2023)	EEG	*N* = 8; more (n_group = 8), n/s, instructor-student	Ecologically valid setting	High	Open-ended	Mixed	[“FTF,” “virtual”]	[“Shared physical IM and verbal IM,” “separate digital IM and verbal IM”]	Temporal domain frequency	[“Social cognition,” “executive function”]
(Chuang et al. 2024)	EEG	*N* = 58; dyad, [“ff,” “fm,” “mm”], n/s	Cooperation/competition tasks	High	Goal-driven	Digital	Virtual	Separate digital IM w/out verbal IM	[“Connectivity functional,” “temporal domain time,” “temporal domain frequency”]	[“Memory,” “visuospatial cognition”]
(Ciaramidaro et al. 2024)	EEG	*N* = 32; dyad, mm, n/s	Coordination tasks	High	Goal-driven	Digital	SBS	Shared digital IM w/out verbal IM	[“Spatial domain source,” “connectivity effective,” “temporal domain frequency”]	[“Motor,” “visuospatial cognition”]
(Cross et al. 2022)	EEG	*N* = 40; dyad, [“fm,” “mm”], n/s	Ecologically valid setting	Low	Goal-driven	Mixed	[“SBS,” “virtual”]	[“Shared physical IM and verbal IM,” “separate digital IM and verbal IM”]	Temporal domain frequency	[“Motor,” “executive function”]
(Du et al. 2022)	EEG	*N* = 36; triad, n/s, familiar	[“Ecologically valid setting,” “cooperation/competition tasks”]	High	Mixed	Digital	Virtual	Separate digital IM and verbal IM	[“Connectivity functional,” “temporal domain frequency”]	[“Language,” “attention,” “executive function”]
Flösch et al. (2024a)	EEG	*N* = 32; dyad, [“ff,” “mm”], familiar	Cooperation/competition tasks	High	Goal-driven	Digital	Virtual	Separate digital IM w/out verbal IM	[“Spatial domain sensor,” “temporal domain frequency”]	[“Memory,” “language,” “social cognition,” “executive function”]
(Flösch et al. 2024b)	EEG	*N* = 24; dyad, [“ff,” “mm”], familiar	Cooperation/competition tasks	High	Goal-driven	Digital	Virtual	Separate digital IM w/out verbal IM	Temporal domain time	[“Memory,” “language,” “social cognition,” “executive function”]
(Gumilar et al. 2021)	EEG	*N* = 24; dyad, n/s, unfamiliar	Coordination tasks	High	Goal-driven	Mixed	Virtual	[“Non-verbal IM,” “shared digital IM w/out verbal IM”]	[“Spatial domain source,” “connectivity functional,” “temporal domain frequency”]	[“Motor,” “visuospatial cognition”]
(Hayati et al. 2025)	EEG	*N* = 28; dyad, [“ff,” “fm,” “mm”], familiar	Cooperation/competition tasks	High	Goal-driven	Mixed	[“SBS,” “virtual”]	Shared digital IM and verbal IM	[“Connectivity functional,” “spatial domain sensor”]	Visuospatial cognition
(Liu et al. 2024)	EEG	*N* = 104; dyad, [“ff,” “mm”], unfamiliar	Cooperation/competition tasks	High	Goal-driven	Digital	SBS v-b	Separate digital IM w/out verbal IM	[“Temporal domain frequency,” “connectivity functional,” “spatial domain source”]	Social cognition
(Léné et al. 2021)	EEG	*N* = 46; dyad, n/s, unfamiliar	Cooperation/competition tasks	High	Goal-driven	Digital	SBS v-b	Shared digital IM w/out verbal IM	[“Connectivity functional,” “temporal domain frequency”]	[“Attention,” “executive function”]
(Wikström et al. 2022)	EEG	*N* = 42; dyad, [“ff,” “fm,” “mm”], familiar	Cooperation/competition tasks	Low	Goal-driven	Digital	virtual	Shared digital IM w/out verbal IM	#BEZUG!	[“Motor,” “attention,” “executive function,” “visuospatial cognition”]
(Zhang et al. 2019)	EEG	*N* = 74; dyad, [“ff,” “mm”], unfamiliar	Cooperation/competition tasks	High	Goal-driven	Digital	FTF v-b	Separate digital IM w/out verbal IM	[“Connectivity functional,” “temporal domain time,” “spatial domain sensor,” “spatial domain source”]	[“Social cognition,” “executive function”]
(Zhou et al. 2021)	EEG	*N* = 60; dyad, [“ff,” “mm”], n/s	Cooperation/competition tasks	High	Goal-driven	Digital	SBS v-b	Separate digital IM w/out verbal IM	#BEZUG!	Motor
**fNIRS**										
(Balters et al. 2023)	fNIRS	*N* = 72; dyad, [“ff,” “fm,” “mm”], unfamiliar	Ecologically valid setting	High	[“Open-ended,” “goal-driven”]	[“analog,” “digital”]	Virtual	Verbal IM	[“Connectivity functional,” “machine learning unsupervised,” “spatial domain sensor”]	[“Social cognition,” “executive function”]
(Cheng et al. 2019)	fNIRS	*N* = 62; dyad, [“ff,” “fm”], unfamiliar	Coordination tasks	High	Goal-driven	Digital	FTF v-b	Separate digital IM w/out verbal IM	[“Connectivity effective,” “connectivity functional”]	[“Motor,” “visuospatial cognition”]
(Hayne et al. 2023)	fNIRS	*N* = 84; dyad, fm, unfamiliar	Cooperation/competition tasks	High	Goal-driven	Digital	Virtual	Separate digital IM w/out verbal IM	[“Spatial domain source,” “machine learning supervised”]	Social cognition
(Hu et al. 2017)	fNIRS	*N* = 70; dyad, ff, unfamiliar	Coordination tasks	High	Goal-driven	Digital	FTF v-b	Separate digital IM w/out verbal IM	[“Spatial domain sensor,” “connectivity functional”]	Social cognition
(Liu et al. 2019)	fNIRS	*N* = 42; dyad, [“ff,” “mm”], instructor-student	Ecologically valid setting	Low	Open-ended	Digital	[“FTF,” “virtual”]	[“Non-verbal IM,” “separate digital IM w/out verbal IM”]	[“Temporal domain time,” “connectivity functional,” “spatial domain sensor”]	Social cognition
(Lu et al. 2020)	fNIRS	*N* = 54; dyad, [“ff,” “mm”], unfamiliar	Ecologically valid setting	High	Open-ended	Mixed	[“FTF,” “virtual”]	[“Verbal IM,” “separate digital IM w/out verbal IM”]	[“Temporal domain frequency,” “connectivity functional,” “spatial domain sensor”]	[“Language,” “social cognition”]
(Pan et al. 2017)	fNIRS	*N* = 98; dyad, fm, [“familiar,” “unfamiliar”]	Coordination tasks	High	Goal-driven	Digital	SBS v-b	Separate digital IM w/out verbal IM	[“Temporal domain frequency,” “connectivity functional,” “connectivity effective”]	[“Motor,” “social cognition”]
(Wu et al. 2025)	fNIRS	*N* = 72; dyad, [“ff,” “fm,” “mm”], unfamiliar	Ecologically valid setting	High	Open-ended	Mixed	[“FTF,” “SBS v-b,” “virtual”]	[“Shared physical IM and verbal IM,” “separate digital IM w/out verbal IM,” “separate digital IM w/out verbal IM”]	[“Temporal domain time,” “spatial domain sensor,” “connectivity functional,” “temporal domain frequency”]	Other
(Yamaya et al. 2025)	fNIRS	*N* = 60; dyad, [“ff,” “mm”], familiar	Coordination tasks	High	Goal-driven	Mixed	[“FTF,” “virtual”]	Verbal IM	Spatial domain source	Language
(Zhang et al. 2023b)	fNIRS	*N* = 480; more (n_group = 6), [“ff,” “fm,” “mm”], unfamiliar	[“Economic exchange tasks,” “cooperation/competition tasks”]	[“High,” “low”]	Goal-driven	Digital	Virtual	Separate digital IM w/out verbal IM	[“Connectivity functional,” “temporal domain frequency,” “spatial domain source”]	[“social cognition,” “executive function”]
(Zhang et al. 2023a)	fNIRS	*N* = 84; dyad, [“ff,” “mm”], unfamiliar	Cooperation/competition tasks	Low	Goal-driven	Mixed	[“SBS,” “SBS v-b”]	Shared digital IM and verbal IM	[“Connectivity functional,” “spatial domain sensor,” “machine learning supervised”]	[“Memory,” “language”]
**Eye-tracking**										
(Cheng et al. 2022)	Eye-tracking	*N* = 32; dyad, n/s, n/s	Ecologically valid setting	High	Goal-driven	Digital	Virtual	Separate digital IM and verbal IM	Other	[“Attention,” “social cognition,” “executive function,” “visuospatial cognition”]
(Findik-Coşkunçay and Çakir 2022)	Eye-tracking	*N* = 27; triad, n/s, n/s	Ecologically valid setting	High	Goal-driven	Digital	Virtual	Separate digital IM w/out verbal IM	[“Temporal domain time,” “connectivity functional”]	[“Attention,” “social cognition,” “executive function”]
(Hoffmann et al. 2024)	Eye-tracking	*N* = 74; dyad, n/s, familiar	Ecologically valid setting	High	Goal-driven	Digital	SBS v-b	Separate digital IM and verbal IM	Connectivity functional	Attention
(Kütt et al. 2019)	Eye-tracking	*N* = 40; dyad, n/s, [“familiar,” “unfamiliar”]	Ecologically valid setting	High	Mixed	Digital	BTB	Separate digital IM and verbal IM	Other	[“Attention,” “language”]
(Pöysä-Tarhonen et al. 2021)	Eye-tracking	*N* = 4; dyad, [“ff,” “mm”], familiar	Cooperation/competition tasks	High	Goal-driven	Digital	Virtual	Separate digital IM and verbal IM	Other	[“Attention,” “social cognition,” “executive function”]
(Wisiecka et al. 2023)	Eye-tracking	*N* = 54; dyad, [“ff,” “mm”], n/s	Cooperation/competition tasks	High	Goal-driven	Digital	[“SBS,” “virtual”]	[“Shared digital IM and verbal IM,” “separate digital IM and verbal IM”]	Other	[“Social cognition,” “executive function”]
Špakov et al. (2019)	Eye-tracking	*N* = 40; dyad, fm, [“familiar,” “unfamiliar”]	Cooperation/competition tasks	Low	Goal-driven	Digital	[“FTF v-b,” “virtual”]	Separate digital IM and verbal IM	Other	[“Attention,” “social cognition,” “executive function,” “visuospatial cognition”]
**Physiological data (ECG, EDA, EMG, other)**										
(Fang et al. 2022)	ECG	*N* = 24; more (n_group = 6), fm, n/s	Cooperation/competition tasks	Low	Mixed	Mixed	[“FTF,” “virtual”]	[“Verbal IM,” “separate digital IM and verbal IM”]	[“Temporal domain time,” “connectivity functional”]	[“Attention,” “social cognition,” “executive function”]
(Strang et al. 2014)	ECG	*N* = 80; dyad, [“ff,” “mm”], unfamiliar	Cooperation/competition tasks	Low	Goal-driven	Digital	FTF	Separate digital IM w/out verbal IM	[“Temporal domain time,” “connectivity functional”]	[“Executive function,” “visuospatial cognition”]
(Le Bars et al. 2020)	EDA	*N* = 35; dyad, [“ff,” “mm”], unfamiliar	Cooperation/competition tasks	Low	Goal-driven	Digital	SBS v-b	Separate digital IM w/out verbal IM	Temporal domain time	[“Executive function,” “visuospatial cognition”]
(Melendez-Calderon et al. 2015)	EMG	*N* = 10; dyad, [“ff,” “mm”], unfamiliar	Coordination tasks	High	Goal-driven	Digital	SBS v-b	Separate digital IM w/out verbal IM	[“Other,” “temporal domain time”]	Motor
(Konvalinka et al. 2023)	Other	*N* = 24; dyad, [“ff,” “fm”], unfamiliar	[“Coordination tasks,” “imitation tasks”]	[“High,” “low”]	Goal-driven	Digital	Virtual	separate digital IM w/out verbal IM	[“Temporal domain frequency,” “connectivity functional,” “temporal domain time”]	Motor

Overview of all included sources and assigned category labels. BTB, back-to-back; ECG, electrocardiography; EDA, electrodermal activity; EEG, electroencephalography; ff, female-female pairing; fm, female-male or otherwise mixed-gender pairing; fNIRS, functional near-infrared spectroscopy; FTF v-b, face-to-face with visual barriers; FTF, face-to-face; IM, interaction medium; mm, male-male pairing; n/s, not specified; SBS v-b, side-by-side with visual barriers; SBS, side-by-side.

### 3.2 Study categorization

In the following section, the results of the categorization of the selected studies according to the labels described in Section 2.4 and detailed in Table 6 are summarized.

#### 3.2.1 Measurement modalities

**Figure 4:**
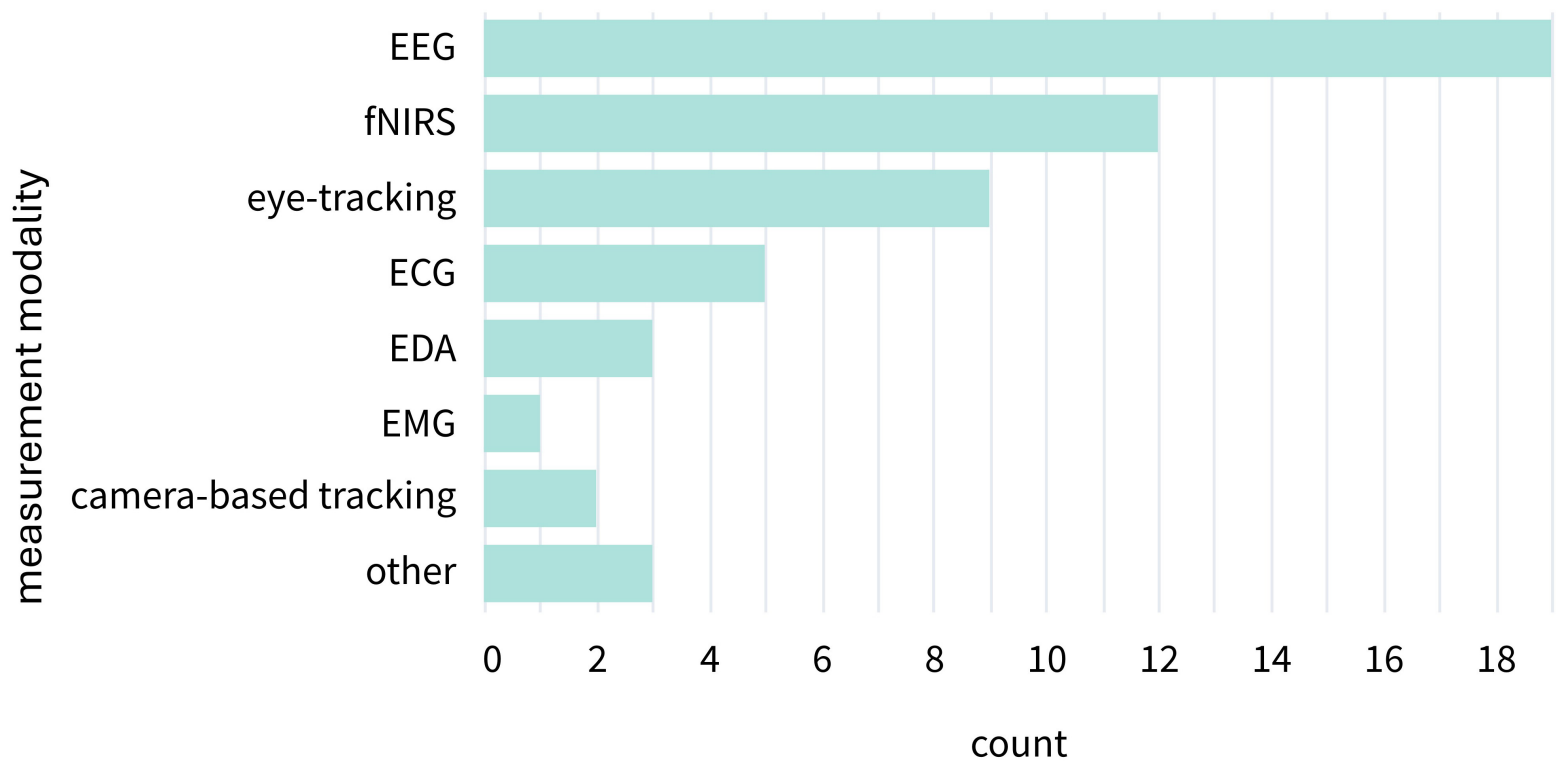
Category counts of measurement modalities. Measurement modalities were distributed across eight categories. The category “other” included two studies measuring respiration and one study measuring impedance cardiography.

Overall, 19 studies included EEG, 12 studies included fNIRS, and the 14 remaining studies included a type of physiological measurement or camera-based tracking of a physiological signal (see Figure 4). Few papers (n = 7) reported results of multiple measurement modalities jointly, and even fewer (n = 5) reported a combination of physiological (body) and neurophysiological (brain) methods (EEG: Balconi et al., 2022; Chuang and Hsu, 2023; Gugnowska et al., 2022; Wang et al., 2024; fNIRS: Shih et al., 2024). With respect to other modalities, eye-tracking was employed in nine studies, but only two studies combined eye-tracking with EEG (Chuang and Hsu, 2023; Wang et al., 2024), and none reported the joint use of fNIRS and eye-tracking. A similarly limited pattern was observed with ECG and EDA: two studies employed ECG alone (Fang et al., 2022; Strang et al., 2014), while EDA was used in only one study as a standalone measure (Le Bars et al., 2020). ECG and EDA were only combined once with EEG (Balconi et al., 2022). Camera-based tracking was integrated with fNIRS in one instance (Shih et al., 2024) and with EEG in another (Gugnowska et al., 2022).

#### 3.2.2 Participants

The majority of studies investigated inter-subject dynamics in a dyadic setting (*n* = 39) with an average sample size of 46.6 ± 26.1 (SEM = 4.17, min: 4, max: 104), although three studies measured triads (Du et al., 2022; Findik-Coşkunçay and Çakir, 2022; Snijdewint and Scheepers, 2023), two studies measured groups of six (Fang et al., 2022; Zhang et al., 2023b), and one paper reported measuring eight individuals at once (Balconi et al., 2023).

Most reported that paired participants were unfamiliar with each other (*n* = 23), although many also used familiar pairings of participants (*n* = 14). Ten studies did not state conclusively whether interacting participants knew each other. In three studies, the relationship between partners was identified to be of an instructor-student nature (Balconi et al., 2022, 2023; Liu et al., 2019).

The majority of studies included measurements of same-sex pairs (31 studies), with one study including only female pairs (Hu et al., 2017) and three studies including only male pairs (Astolfi et al., 2020; Ciaramidaro et al., 2024; Numata et al., 2021). Fewer studies indicated a setup with different-sex pairs (*n* = 17), with four studies including only mixed-gender pairs (Fang et al., 2022; Hayne et al., 2023; Pan et al., 2017; Špakov et al., 2019). For 14 studies, the combination of pairs was not specified conclusively. Note that the number of participants who indicated to identify as “non-binary” or “other” was negligibly small, although in this case, the setup was counted as a different-sex setup (Fang et al., 2022; Hayne et al., 2023; Špakov et al., 2019).

#### 3.2.3 Paradigm category

We adapted a paradigm categorization based on six previously defined archetypes (Barde et al., 2020; Wang et al., 2018). The paradigm categorization revealed that for the studies included in our review, most studies represented cooperation/competition tasks (*n* = 22), while some paradigms were more representative of an ecologically valid setting (*n* = 16). Seven studies were identified as belonging to the category of coordination tasks. One study employed a cooperation/competition paradigm with an interaction mechanic relying on gaze itself (Chuang and Hsu, 2023). In another study, respiratory synchrony in a coordination task was compared to that in an imitation paradigm (Konvalinka et al., 2023). The latter study used a confederate who did not actually collaborate, yet it was included because participants believed they were engaging in a collaborative task related to respiration coordination. Most studies utilizing a form of economic exchange tasks were not included since they lacked a collaborative component (i.e., focused on competitive interactions), except one study in which participants jointly participated in such a task to investigate collaborative decision making in competing groups (Zhang et al., 2023b).

#### 3.2.4 Task symmetry

Thirty-three studies involved high task symmetry between participants, i.e., participants fulfilling similar roles, whereas 14 studies were classified as low-symmetry studies, i.e., they involved predefined, distinct roles. One study incorporated a range of tasks with varying symmetry (Konvalinka et al., 2023), and another involved the simultaneous measurement of multiple participants, some exhibiting high symmetry and others low symmetry toward each other (Zhang et al., 2023b).

#### 3.2.5 Type of communication

To better understand the nature of interaction in hyperscanning studies, we examined the objective of communication. Goal-driven communication was assumed when an explicit goal, objective or outcome was pursued in the task. Any task with no clear or explicit objective was categorized as open-ended. Tasks containing elements of both were defined as mixed communication. Thirty-three studies involved goal-driven communication (either verbal or non-verbal). Nine studies involved open-ended communication, of which all were identified as utilizing an ecologically valid setting during the sessions. Four studies employed a mixed communication (Du et al., 2022; Fang et al., 2022; Kütt et al., 2019; Numata et al., 2021). For instance, one study had participants play a game of *Mafia*, which allows for an open-ended portion of communication as part of the game, where participants could freely choose whether to speak and what to say (Fang et al., 2022).

#### 3.2.6 Transfer of information

Before detailing the transfer of information based on the interaction medium and the interaction scenario, we broadly categorized whether information exchange between participants happened via analog, digital, or mixed channels (Balters et al., 2020). Since one of the study eligibility criteria was the focus on a digital aspect in collaboration, all included studies utilized some form of digital information transfer, where most of them (*n* = 34) examined exclusively digital communication. Approximately a fourth of all studies (*n* = 11) involved a mixed information transfer of both digital and analog ways. One study included a between-subjects comparison of remote and in-person collaboration and hence was also categorized as containing an analog form of information transfer (Balters et al., 2023).

#### 3.2.7 Interaction scenarios

Interaction scenarios refer to the physical or virtual setup of the participants relatively to each other, e.g., facing each other, sitting side-by-side, or interacting remotely via screens or Virtual Reality setups. As expected, based on the search criteria, most studies took place in a form of virtual scenario (*n* = 29). Some approximated a remote setting by placing a visual barrier between participants positioned side-by-side (*n* = 10), face-to-face (*n* = 6), or seating them back-to-back (*n* = 1). Some studies directly compared one of these scenarios to a collaborative interaction in person or utilized a form of purely digital means of interaction medium, leading to the inclusion of face-to-face (*n* = 9) and side-by-side (*n* = 6) paradigm scenarios.

#### 3.2.8 Interaction medium

Interaction medium refers to the kind of interaction that the participants experience with each other and what type of medium was used, e.g., whether participants were allowed to verbally communicate, whether they interacted with physical objects, or via a digital tool (see Table 4). Most studies utilized a separate means of digital interaction (e.g., two screens) without verbal exchanges (*n* = 24). A few had similar setups while allowing for verbal interactions (*n* = 13). Fewer studies utilized shared digital interaction media (without verbal: *n* = 4; with verbal: *n* = 3).

#### 3.2.9 Comparison of the interaction medium and scenario

**Figure 5:**
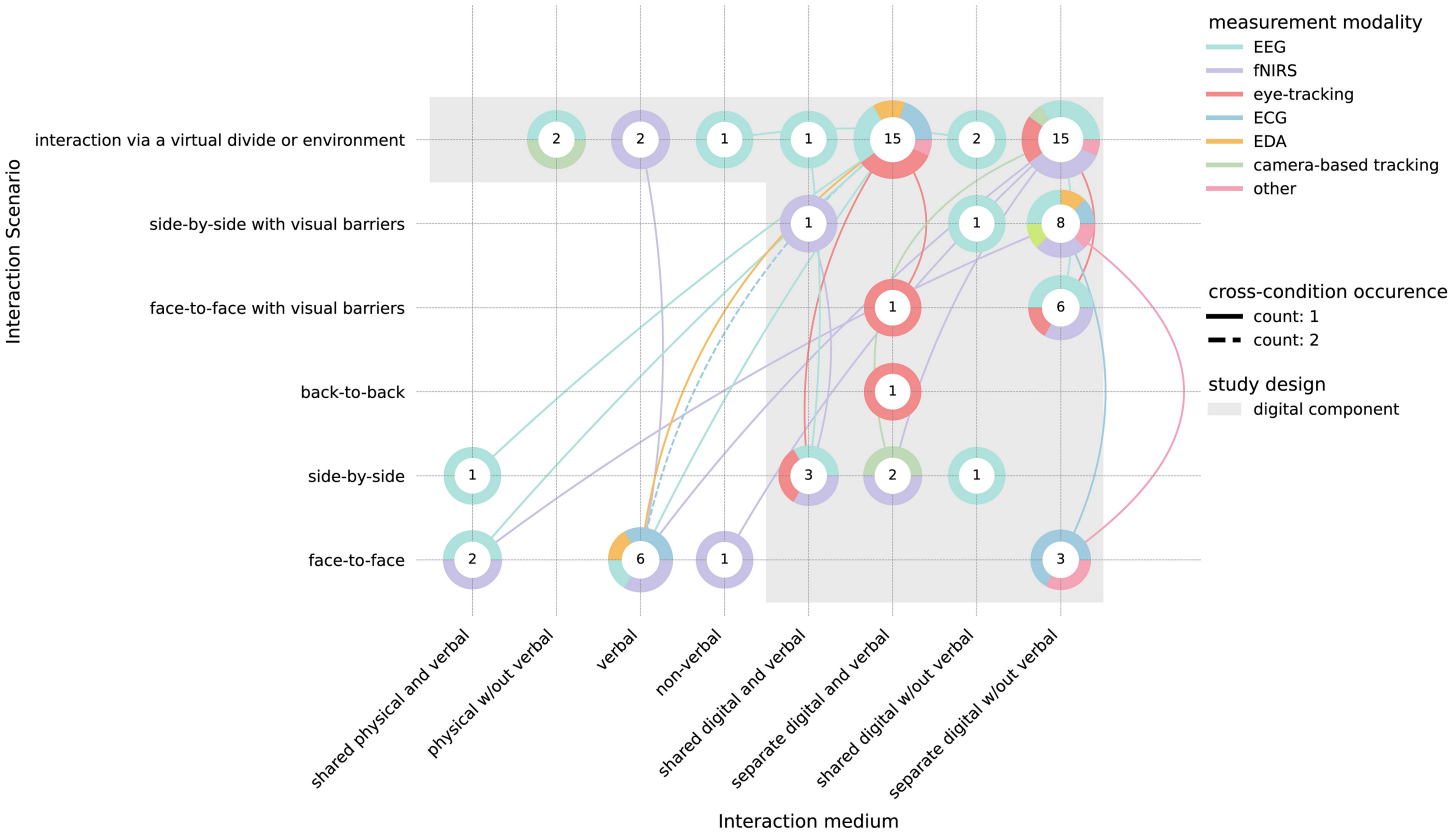
Distribution of hyperscanning conditions across modalities and interaction types. The cross-sectional distribution of all hyperscanning conditions of 45 studies across interaction medium and interaction scenario axes. The numbers in circles provide the counted occurrences (*n* = 75 conditions across modalities) of the cross-section of an interaction medium and scenario (*n* = 22 unique combinations, shown as circles). The colors represent the measurement modalities reported for each cross-section of conditions. The connection lines indicate reported cross-condition occurrences separated by axis (*n* = 16 simultaneous condition occurrences). Studies involving a digital component, either through a digital medium or a virtual interaction scenario, are marked through a gray shaded area. Note that conditions that do not fall within this gray area are part of a comparison to a condition with a digital component. ECG, electrocardiography; EDA, electrodermal activity; EEG, electroencephalography; fNIRS, functional near-infrared spectroscopy.

We also looked at the interaction medium and the interaction scenario jointly to define the extent to which a study design is digital (Balters et al., 2021). For a differentiated analysis, we mapped studies along the dimensions of interaction medium, including the presence of verbal communication, interaction scenario, measurement modality, and cross-condition occurrences within the same study. Results are summarized in Figure 5 and Supplementary Table 2.

Across 45 multimodal hyperscanning studies, a total of 75 experimental conditions were reported, consisting of 22 unique combinations of interaction medium and interaction scenario (see Figure 5). Moreover, 16 co-occurrences of two or more conditions comparing medium and/or scenario were found, with eight comparisons of a digital with a non-digital interaction setup (for details see Supplementary Table 2). The number of studies including non-verbal communication (*n* = 26) was slightly higher than the number including verbal communication (*n* = 19). Notably, most studies were designed with a virtual divide or environment and separate interaction media, independent of the inclusion of verbal communication.

Some modality-specific patterns became evident by examining the cross-sectional distribution of interaction medium and scenario variations (for details, see Supplementary Table 2). FNIRS studies included more occurrences of various interaction conditions within the same study, including several that contrasted physical, hybrid, and fully digital interactions, as well as the greatest relative number of unique medium-scenario combinations (*n* = 10 in 12 included studies) with six cross-condition occurrences within the same studies (for details, see Supplementary Figure 2). EEG studies also included a high number of unique medium-scenario combinations (*n* = 14 in 19 included studies), however, only six studies included more than one condition at once (for details, see Supplementary Figure 3). Eye-tracking studies were confined to digital scenarios, resulting in fewer unique combinations and no direct digital-analog comparisons (for details, see Supplementary Figure 4). Together, these patterns highlight substantial methodological variation across modalities in how interaction settings are operationalized and compared.

#### 3.2.10 Analysis approaches

**Figure 6:**
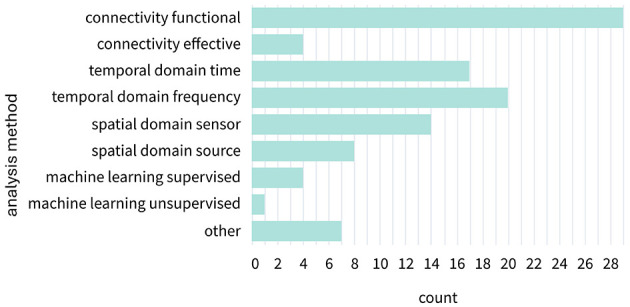
Category counts of analysis methods. Analysis methods were distributed across eight categories. The category “other” included two studies assessing the amount of gaze-sharing, two studies analyzing gaze patterns, and one study measuring muscle co-contraction.

The most common analysis method involved measuring functional connectivity (*n* = 29), with some studies also including effective connectivity (*n* = 4). Of these, two used Granger causality (Cheng et al., 2019; Pan et al., 2017) and two computed partial directed coherence (Astolfi et al., 2020; Ciaramidaro et al., 2024). The second most prevalent signal analysis approach focused on the temporal domain, examining time (*n* = 17) or frequency (*n* = 20) aspects. A few studies included analyses in the spatial domain, be it in sensor space (*n* = 14) or source space (*n* = 8). Two EEG studies (Astolfi et al., 2020; Zhou et al., 2021) and two fNIRS studies (Hayne et al., 2023; Zhang et al., 2023a) employed a form of supervised machine learning. One study employed a support vector machine (SVM) to differentiate between cooperative, solo, and competitive gaming based on hemodynamic features from executive and motor regions (Hayne et al., 2023). Notably, they determined within-subject features during the cooperative interaction rather than features based on dyadic indices. Another fNIRS study predicted mnemonic similarity based on the interpersonal neural synchronization during a collaborative remembering task (Zhang et al., 2023a). By creating features based on graph indices derived from partial directed coherence values, social and non-social conditions were distinguished using an SVM. (Zhou et al. 2021) used a logistic regression to demonstrate that the conditional manipulation of dyads could be predicted by using cumulative inter-brain synchrony from significant electrode pairs as classification features. Only one fNIRS study reported the use of unsupervised machine learning to investigate interactions, namely a k-means clustering approach to identify dynamic interbrain coherence states along with their corresponding occurrence rates across time during an online conversation task (see Figure 6; Balters et al., 2023). Other analysis methods included a form of graph theory (Astolfi et al., 2020), two reports on the amount of gaze sharing (Kütt et al., 2019; Wisiecka et al., 2023), another three focusing on gaze pattern analysis (Cheng et al., 2022; Pöysä-Tarhonen et al., 2021; Špakov et al., 2019), and one study analyzing co-contraction of two muscles in the right forearm measured via EMG (2015).

#### 3.2.11 Cognitive functions of interest

**Figure 7:**
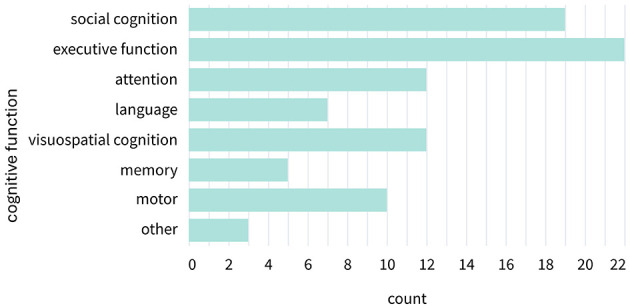
Category counts of cognitive functions. Cognitive functions were distributed across eight categories. The category “other” included two studies focusing on joint designing ability and one study assessing joint musical ability.

Categorization of the cognitive function of interest was not clear-cut because many tasks required the engagement of multiple cognitive functions. Based on explicitly stated research objectives (e.g., Zhang et al., 2023a) or the described tasks (e.g., a visual search task; Szymanski et al., 2017), the main functions of interest were categorized (see Figure 7). Perhaps unsurprisingly, given the focus on collaborative interactions, most studies focused on executive functioning (*n* = 22) or social cognition (*n* = 19). Various studies addressed visuospatial cognition (*n* = 12) and attention (*n* = 12) during collaborative scenarios. Some studies focused, by design, more strongly on motor functions (*n* = 10) or language (*n* = 7). Few studies mainly investigated memory-related processes (*n* = 5) or other cognitive functions (*n* = 3). The latter included two studies that emphasized creative design (Shih et al., 2024; Wu et al., 2025) and one focusing on joint musical ability during remote piano playing (Gugnowska et al., 2022).

#### 3.2.12 Relating modality, analysis, and cognitive function

**Figure 8:**
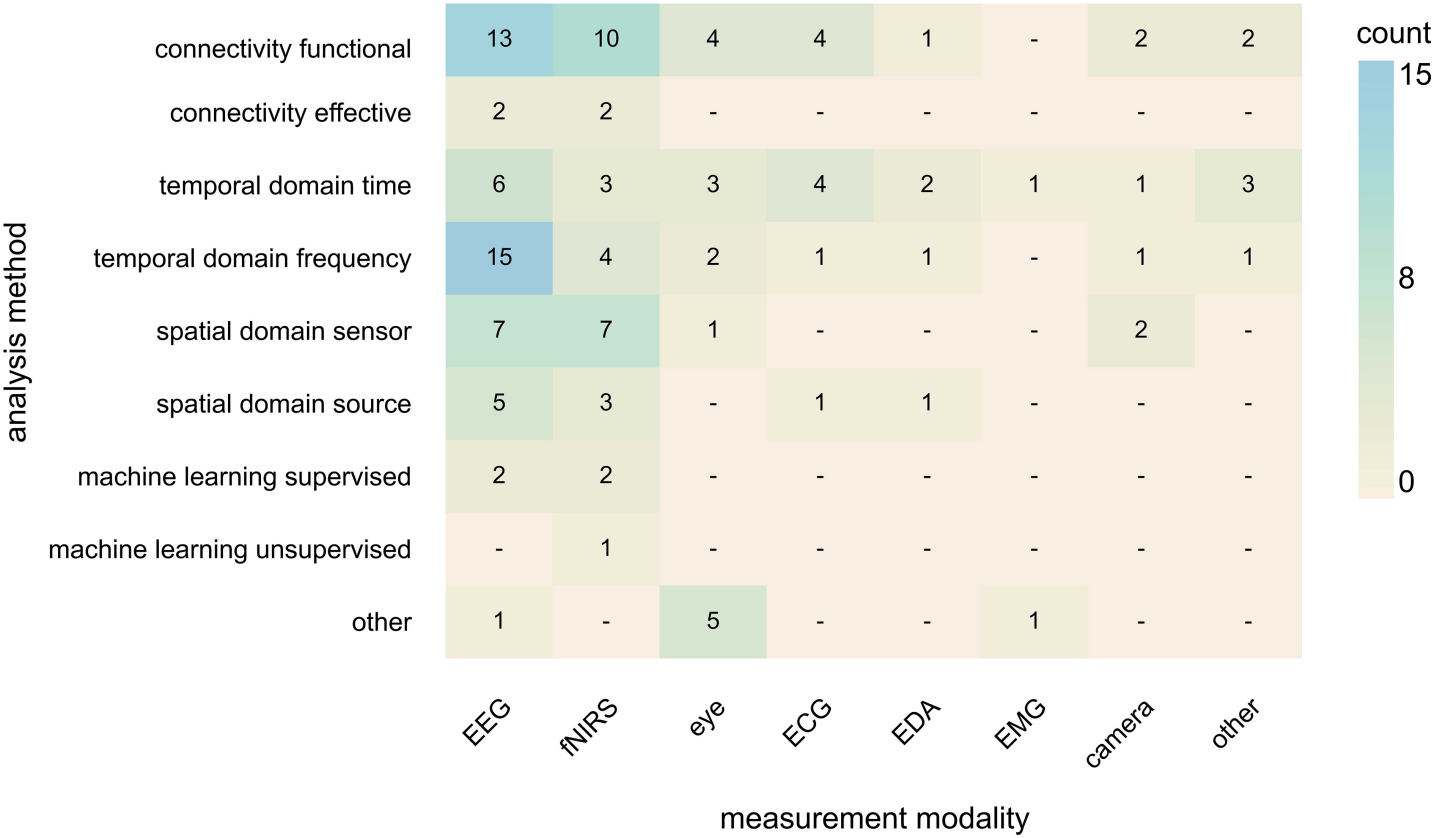
Heatmap of measurement modalities and analysis methods. Heatmap of studies across the categorical dimensions analysis method (rows) and measurement modality (columns). Cell values show raw co-occurrence counts between the row and column categories. Cell color intensity increases with the value in each cell; darker cells indicate higher numbers. camera, camera-based tracking; ECG, electrocardiography; EDA, electrodermal activity; EEG, electroencephalography; EMG, electromyography; eye, eye-tracking; fNIRS, functional near-infrared spectroscopy.

**Figure 9:**
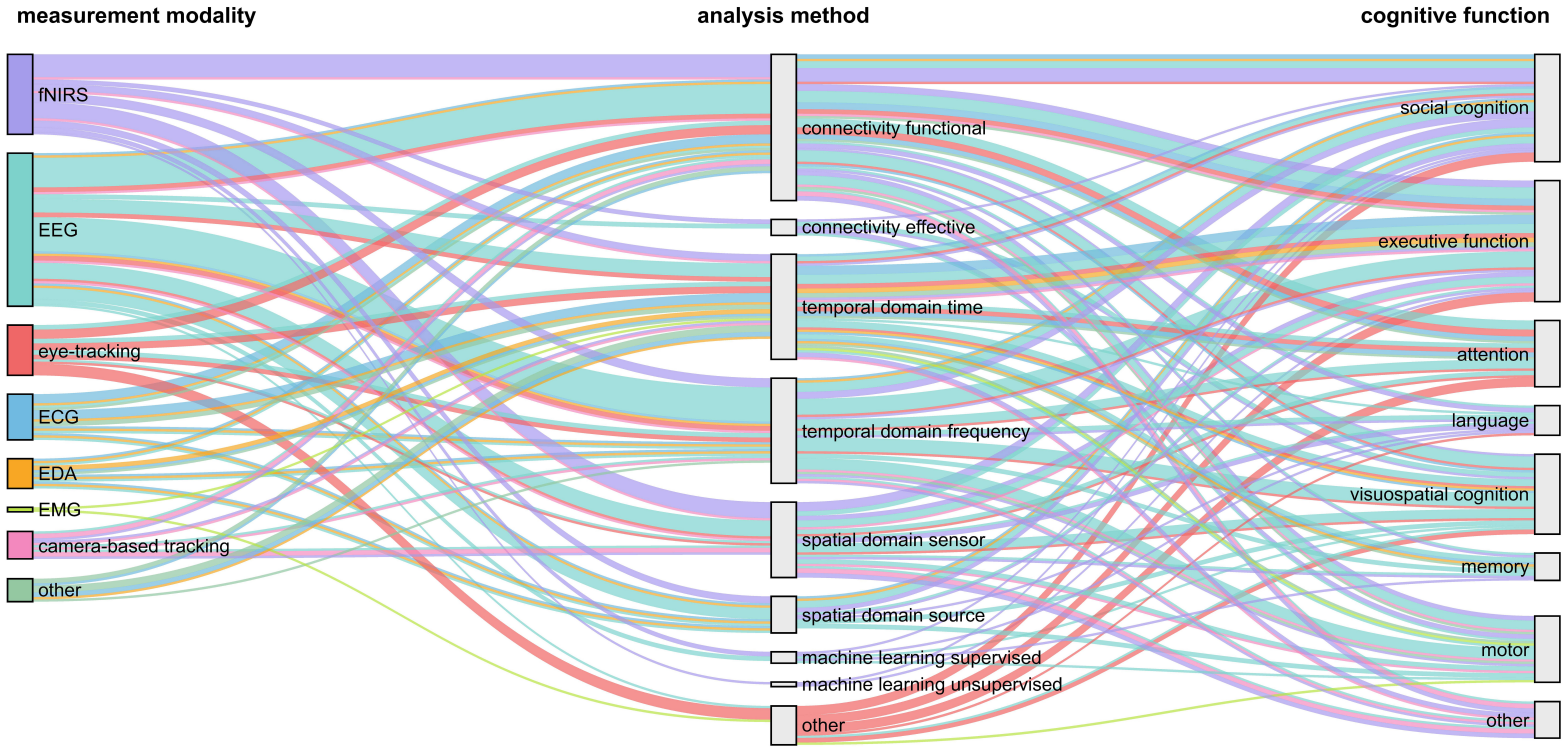
Alluvial plot of measurement modality, analysis method, and cognitive function. Alluvial diagram illustrating category flows across the categories measurement modality (left), analysis method (middle), and cognitive function (right) shown as vertical columns of nodes. Node height is proportional to the number of outgoing links summed across all destinations, and link thickness reflects transitions between adjacent categories. Colors indicate measurement modality. Note that some nodes in the diagram originate from one measurement modality but are colored according to another modality to represent studies in which multiple measurement modalities were used simultaneously.

To understand how measurement modality aligns with analysis and cognitive function under study, we plotted their relation in Figures 8, 9. These comparisons highlight clear patterns in analytic preferences as well as notable methodological gaps.

Overall, fNIRS emerged as the most versatile modality regarding analysis, appearing across nearly all analysis categories. It also represented the only instance of unsupervised machine learning within the included studies. While EEG was the most prominently used modality, studies showed a more selective analysis profile, with analyses predominantly situated in the functional connectivity and the frequency domain. Eye-tracking studies were distributed in nearly equal proportions across time-domain, functional connectivity approaches, and other forms of analyses. Peripheral physiological signals (e.g., ECG, EDA, EMG) were primarily analyzed in the time domain and through functional connectivity approaches, reflecting an application in capturing autonomic or muscular synchrony. Camera-based tracking appeared only sparsely across several categories. Across modalities, several analytic approaches appeared markedly underutilized. Effective connectivity was rare, with only a handful of studies applying it despite its relevance for examining directional influences between interacting partners (Astolfi et al., 2020; Cheng et al., 2019; Ciaramidaro et al., 2024; Pan et al., 2017). Machine learning approaches were equally rare (Astolfi et al., 2020; Balters et al., 2023; Hayne et al., 2023; Zhang et al., 2023c; Zhou et al., 2021), with only one study employing an unsupervised approach (Balters et al., 2023). Both effective connectivity and machine learning methods were limited to brain-based modalities. Together, these patterns indicate a strong reliance on functional connectivity and temporal analyses, with substantial methodological opportunities for future work.

Across modalities and analytic approaches, executive function and social cognition consistently emerge as the most frequently investigated cognitive domains, forming the largest group of targeted cognitive functions. Executive function was associated with the largest body of studies overall, particularly from EEG-based time-domain, frequency-domain, and functional connectivity analyses, as well as from fNIRS connectivity approaches. Social cognition exhibits an equally robust presence, especially in fNIRS studies employing functional connectivity methods as well as in eye-tracking studies. In contrast, domains such as memory, language, and motor cognition were addressed in fewer studies. Visuospatial cognition occupies a middle ground: although not as prominent as executive or social cognition, it shows a clear association with EEG-based temporal and spatial analyses, indicating a more specialized but stable niche. Interestingly, comparatively few studies involving eye-tracking focused on visuospatial cognition (for details, see Supplementary Figure 5).

## 4 Discussion

We systematically collected and analyzed studies on hyperscanning that explored collaboration in digital contexts. The categorization revealed that most studies used either EEG or fNIRS as measurement tools, with only seven out of 45 employing multimodal measurement approaches. Among these, most combined EEG with either eye-tracking or ECG, indicating a notable underuse of multimodal measurement techniques. Most studies focused on same-sex dyads, with roughly two-thirds of participants unfamiliar with each other. The studies mainly involved cooperation or competition tasks or aimed at modeling an ecologically valid setting. Tasks driven by goals with symmetrical roles between participants were most common in examining collaboration in digital environments and media. Interestingly, most studies limited information transfer to digital formats such as screen-based exchanges, with fewer studies utilizing both analog and digital transfer methods. FNIRS studies frequently examined different interaction scenarios or media across various conditions, while EEG studies featured a broader variety of unique interaction settings. Regardless of interaction setup, non-verbal compared to verbal communication was investigated somewhat more often, albeit the difference was small. Analytical approaches were mostly constrained to functional connectivity, with limited application of effective connectivity. Furthermore, very few studies applied machine learning, highlighting a methodological gap given the high-dimensional, complex data involved. Research has primarily concentrated on executive and social functions during digital collaboration, while fewer studies have addressed creativity, memory, or language explicitly. Our thorough review of existing literature indicates a solid foundation of research, although its scope can be extended. To better understand the complex dynamics of digital collaboration, it is essential to expand both the methodological framework and the conceptual basis of hyperscanning research.

Before discussing the most important aspects and main takeaways in detail, we summarize the central conclusions here. First, we recommend a set of reporting guidelines for hyperscanning studies to increase transparency, reproducibility, and meaningful comparison across the literature, including a checklist to help authors document essential methodological information. Second, we emphasize the contribution of the present review and the associated online database in organizing hyperscanning research into standardized categories. Third, based on the diversity of study designs identified, we recommend conducting systematic empirical comparisons of differently structured interaction scenarios to support the development of theories and the application of findings to real-world settings. Fourth, our results show that the unimodal use of measurement modalities currently dominates the hyperscanning research on digital collaboration. However, we identify considerable potential in integrating multiple modalities to construct multidimensional models of the neurophysiological signatures of collaboration. Such approaches could provide complementary perspectives on collaborative cognition and how digital components influence associated cognitive and social processes. Fifth, the present review also suggests that the choice of measurement modality is strongly associated with not only the analytic approach but also the specific cognitive processes investigated. We therefore argue that multivariate and multimodal approaches will be necessary to disentangle the involvement of the many cognitive processes involved in collaborative behavior, and to capture fine-grained effects associated with different degrees of digital mediation. Finally, we encourage readers and researchers to increase the value of this work by contributing additional sources to the online database, thereby supporting the continued comprehensiveness of the InterBrainDB on digital collaboration.

### 4.1 Structuring principles: guidelines for reporting hyperscanning results

To promote transparency, reproducibility, and meaningful comparison across hyperscanning studies, we strongly encourage authors to report specific methodological details that directly impact the interpretation of inter-brain connectivity and synchronization. Based on our review of the literature, we outlined a set of minimal reporting guidelines that, if followed, greatly improve the interpretability and meta-analytic usefulness of future research (see Table 7).

**Table 7:** Guidelines for reporting hyperscanning findings.

**Category**	**Checklist item**
Sample size	Number of participants or pairings included in analyses (i.e., not necessarily how many were originally recruited)
Participant metadata	Age of participants
	Gender distribution within sample
	Familiarity or relationship between participants (e.g., friends, parent-child)
	Pairing characteristics (e.g., dyads, triads)
	Gender pairing setup (e.g., same-sex pairs)
	Relevant participant traits (e.g., handedness, prior experience)
Measurement modality	Type and technical details of measurements (e.g., EEG, number of electrodes)
	Use and joint analysis of multiple modalities (e.g., EEG and eye-tracking)
Recording configuration	Timing of neural recordings (e.g., simultaneous, post hoc sync)
Communication parameters	Allowed communication types (e.g., verbal, gesture)
	Form of interaction (e.g., open-ended conversation, face visibility)
Task design and structure	Spatial setup (e.g., face-to-face, side-by-side) with diagram if possible
	Interaction media (e.g., shared screen, physical objects)
	Task symmetry (e.g., identical vs. distinct roles)
	Task paradigm (e.g., cooperation, imitation, economic exchange)
Analysis	Level of analysis (e.g., intra-brain, inter-brain) with clear separation in the methods section

This checklist summarizes recommended reporting practices for hyperscanning studies to improve transparency, reproducibility, and comparability across experiments. Items are grouped by major methodological domains, including participant characteristics, measurement modalities, recording configuration, communication parameters, task design, and analytic levels. Examples in parentheses illustrate typical reporting details but are not exhaustive.

Firstly, researchers should thoroughly report participant metadata. This includes age, gender, familiarity among participants, and any pairing characteristics that might systematically influence neural coupling or social behavior (e.g., handedness, prior experiences). Studies that do not disclose this information limit the ability to meaningfully contextualize, compare, or replicate their results.

Secondly, it is crucial to specify the temporal setup of the neural recordings, i.e., whether data were recorded simultaneously across participants or if signal streams were aligned afterward. Although both methods are valid, they have different implications for interpreting time-locked neural coupling and inter-brain dynamics. On one side, simultaneous recordings offer higher ecological validity for social components compared to single-person recordings; on the other side, single-person recordings provide greater experimental control and easier replication that is less dependent on the sample (Fan et al., 2021). The included studies used simultaneous acquisition (as part of the *comparison* inclusion criterion). However, during full-text screening, a notable subset (*n* = 46) was excluded because they relied on separate acquisitions with later alignment, even though the abstract screening suggested a form of real-time social interaction.

Thirdly, authors should clearly state whether verbal communication was allowed between participants and, if so, describe the nature and structure of the dialogue. Was communication entirely open-ended, limited to specific prompts, or completely prohibited? The level of verbal interaction fundamentally alters the cognitive and emotional demands of the task (Lu et al., 2020). Apart from affecting social, cognitive, emotional, and sensory processes, it can also introduce signal noise through muscle activity. Studies should specify whether verbal and/or non-verbal communication was possible during the experiment, as both can influence interpersonal neural synchrony in collaborative settings (Shih et al., 2024).

Fourthly, it is essential to describe the visual and spatial arrangement of the participants during the experiment to help readers better understand the sensorimotor and perceptual context of the interaction. Specifically, were participants able to see each other's facial expressions, gestures, and body movements? How were they positioned relative to each other (e.g., face-to-face, side-by-side, separated by a screen)? We recommend including a schematic diagram or photo of the experimental setup whenever possible.

Fifthly, we recommend clearly stating whether analyses were conducted at an intra- or inter-individual level or both. To ensure a structured and clear understanding of the methods, we suggest dividing the description of methods accordingly. For studies involving multimodal data collection, we recommend separating the description of analysis methods by measurement modality and clearly specifying how the modalities were combined and analyzed together.

By following these reporting practices, researchers can ensure their hyperscanning studies are both understandable on their own and useful to the larger research community. Future meta-analyses, database-driven reviews, and cumulative science efforts will depend heavily on the consistency and completeness of this essential information.

### 4.2 Study designs for measuring collaboration in the digital age

The wide variety of how factors relevant for digital collaboration are implemented across hyperscanning studies, whether through virtual interfaces, perceptual barriers, or hybrid setups, illustrates the diversity and adaptability of current research methods. A key contribution of this review is the systematic organization of interaction scenarios and media, offering a clear overview of how researchers define digital collaboration. This categorization is an important initial step toward combining findings on remote collaboration across different modalities. Building on this foundation, future research will benefit from designs that directly compare multiple measurement approaches during various interaction phases, as such methods can help develop comprehensive theoretical models and support applying hyperscanning insights to real-world digital environments (Schneider et al., 2021).

### 4.3 Modality: from unimodal to multimodal hyperscanning in digital collaboration

Across the reviewed literature, distinct (neuro-)physiological modalities were associated with specific aspects of collaborative cognition. This pattern could reflect both methodological affordances and historical trends in the field of hyperscanning. FNIRS studies more often targeted social cognition and language processes compared to EEG, leveraging spatial specificity and relative robustness to motion artifacts to probe regions such as the inferior frontal gyrus or temporoparietal junction (Czeszumski et al., 2022). EEG, by contrast, was predominantly used to study executive functions and visuospatial cognition, leveraging its millisecond-level temporal resolution and sensitivity to oscillatory synchronization across distributed networks. Eye-tracking hyperscanning was used less frequently and primarily to investigate attentional coordination between collaborators, consistent with gaze as a behavioral marker of visual attention (Ferencová et al., 2021). Yet relatively few eye-tracking studies explicitly focused on visuospatial collaborative processes, despite shared digital workspaces being central to many contemporary platforms (see Supplementary Figure 5). This represents a missed opportunity to characterize how gaze alignment supports joint reference, turn-taking, and spatial negotiation in digital environments.

Multimodal approaches remain rare. The most common pairing combined EEG with eye-tracking or ECG, the latter likely reflecting existing EEG measurement infrastructures. Notably, we did not identify any hyperscanning studies that integrated EEG and fNIRS in digital interaction contexts, despite strong theoretical motivation. Such integration could jointly capture the spatial localization and temporal dynamics of inter-brain coupling (for review see Li et al., 2022), offering a more complete description of collaborative processes, especially in more applied, naturalistic contexts (Pinto-Orellana et al., 2024). The infrequent use of multimodal approaches in hyperscanning studies highlights an important area for future research, with the potential to significantly advance methodological rigor and ecological validity in this field.

Multimodal acquisition is not trivial: hardware integration, increased setup complexity and cost, paradigm design that accommodates diverse temporal and spatial constraints, and a lack of standardized pipelines for joint analysis can be challenging (Gado et al., 2023; Li et al., 2022; Pinto-Orellana et al., 2024). Nevertheless, multimodal acquisition can provide a uniquely holistic view of how neural, physiological, and behavioral dynamics systematically interact during collaborative interaction (Balconi and Angioletti, 2023; Gado et al., 2023; Li et al., 2022; Lühmann et al., 2020). Work outside collaborative digital contexts already illustrates this potential (Balconi and Angioletti, 2023; Zhao et al., 2023). For example, joint EEG-fNIRS acquisition during a motor synchronization task yielded complementary inter-brain coherence indices across modalities (Balconi and Angioletti, 2023). Hyperscanning in digital collaboration could benefit from adopting symmetric, unsupervised EEG-fNIRS fusion pipelines (for review see Codina et al., 2025) to capture hidden inter-brain coupling beyond standard functional connectivity metrics.

These examples highlight how multimodal setups can shift interpretation from isolated signals to integrated multidimensional models of collaboration. The scarcity of such approaches in digital collaboration studies indicates substantial untapped potential, especially as hardware synchronization and computational fusion methods advance (Codina et al., 2025; Dissanayake et al., 2025). This is particularly critical in digital contexts, where key interaction channels (e.g., eye contact, gestures, spatial proximity) are filtered, delayed, or absent. Different modalities exhibit varying degrees of sensitivity to diverse stimuli (Stuldreher et al., 2020), making multimodal metrics the most robust choice for studying complex, dynamic, and digitally mediated forms of collaboration.

In sum, future work should move beyond simple one-to-one modality-process mapping. In digitally mediated environments, where cognitive, perceptual, and communicative cues are systematically altered, each modality provides a complementary perspective on collaborative cognition. Designing studies that leverage these strengths in combination, while acknowledging their limitations, will be crucial for advancing the cognitive neuroscience of digital interaction.

### 4.4 Analysis approaches and cognitive targets: how methods shape what we can see

Functional connectivity analyses (e.g., IBS measures) remain dominant as an analytical framework across hyperscanning modalities. This prevalence may reflect both conceptual accessibility and methodological convenience, especially in unimodal setups. Temporal-domain analyses were found to be common as well, especially in EEG, ECG, EDA, and eye-tracking, where high temporal resolution lends itself naturally to event-locked and synchrony-based metrics of coordination and attention aspects of collaboration. On the other hand, fNIRS studies frequently employed spatial-domain analyses to leverage its superior cortical specificity. These modality-aligned analytical preferences collectively restrict the kinds of collaborative processes that can be detected and meaningfully interpreted.

Interestingly, only four out of 45 studies incorporated measures of effective connectivity. A possible explanation could be that effective connectivity measures require explicit biophysical or statistical generative models and thus impose stricter assumptions on the data. Violations of these assumptions can produce misleading causal inferences (Friston, 2011). This makes effective measures harder to interpret and less reliable in dynamic social contexts with shared stimuli and mutual influence. Functional connectivity, on the other hand, is more robust and intuitive for the naturalistic data that increasingly characterize hyperscanning studies.

These methodological constraints have direct implications for the cognitive functions that become tractable targets of investigation. Although many collaborative tasks inherently engage multiple cognitive processes, a consistent pattern emerged: executive functions and social cognition are the primary targets of interest. This emphasis is expected, given their role in coordinating shared goals, managing joint attention, regulating turn-taking, and negotiating decisions during collaboration. More fine-grained characterization of the cognitive dynamics underlying collaboration will likely require multivariate and multimodal methods capable of disentangling overlapping components within complex tasks. Combining complementary modalities with data-driven methods may clarify how specific subprocesses, for example, shared attention, perspective taking, and goal monitoring, unfold over time and how they jointly support digitally mediated collaboration. A fine distinction such as this also holds potential for providing specific feedback on interaction processes that may be diminished or lost when interaction shifts from analog to digital (Chuang and Hsu, 2023; Pöysä-Tarhonen et al., 2021).

Given the limitations of conventional analyses for capturing these complex dynamics, machine learning represents an underutilized but promising avenue for advancing hyperscanning research. One reason for the underuse of machine learning probably lies in the field's early developmental stage: many research groups prioritize traditional statistical approaches to establish basic mechanisms of joint action before introducing more complex computational models that are difficult to interpret. Yet, the promise of machine learning goes beyond classification. Its strength lies in modeling non-linear, high-dimensional, and temporally evolving dependencies across multiple interacting processes. For instance, combining fNIRS and EEG features in intra-brain contexts has been shown to enable classification of cognitive workload via bivariate connectivity metrics, an approach readily adaptable to hyperscanning data (Cao et al., 2022). In digitally mediated environments, where communication channels are constrained or altered by technology, such dynamic, cross-modal modeling may be essential for explaining systematic differences between analog and digital collaboration.

A promising direction involves multimodal, physiology-informed machine learning that jointly integrates neural signals (EEG, fNIRS), autonomic measures (ECG, EDA), behavioral indicators (e.g., gaze, EMG), and contextual information into a shared, multidimensional representational space (Codina et al., 2025). Most current fusion strategies rely on methods such as feature concatenation or decision-level fusion, whereas more powerful approaches (e.g., joint independent component analysis) remain underused despite their ability to reveal latent, cross-domain structure (Codina et al., 2025). Embedding physiological priors, such as neurovascular coupling models or autonomic response patterns, directly into preprocessing and fusion could push hyperscanning beyond simple synchrony indices toward richer, context-aware models of collaborative interaction. Progress in this direction is tightly coupled to the availability of open-source, high-quality datasets. As highlighted in a recent overview of human synchronization datasets by (Velletaz et al. 2025), multimodal data remain scattered and underutilized. Expanding accessible, well-curated datasets will be critical for enabling advanced analytic developments and for refining our understanding of the fine-grained, interacting cognitive processes that support digitally mediated collaboration.

### 4.5 Limitations

Several limitations of the present review should be acknowledged. First, by focusing on collaborative tasks with digital components, we excluded hyperscanning studies that are, for example, centered on imitation, purely economic exchange paradigms, or shared attention. The resulting distribution of paradigms does therefore not reflect the full landscape of hyperscanning research. Nonetheless, even within this constrained scope, we identified several more open-ended and applied designs, suggesting a broader trend toward ecologically valid applications. Second, we deliberately excluded fMRI and MEG to focus on modalities amenable to mobile and applied use. One could argue, however, that fMRI and MEG hyperscanning inherently involves digital elements by design. These modalities are therefore relevant to digital interaction. The associated living database is intended to be extended over time to incorporate such studies, enabling a more comprehensive overview. Notably, although the present review is static, the InterBrainDB platform is designed as a living, open-source resource. New studies can be added and re-labeled over time, and analyses can be replicated with different inclusion filters (e.g., adding modalities or relaxing collaboration criteria). In this way, some of the limitations of the present synthesis, particularly with respect to coverage and modality scope, can be progressively mitigated as the database grows.

## 5 Conclusion

This systematic review synthesizes hyperscanning research on digitally mediated collaboration and reveals a field that is expanding in scope, albeit still constrained by methodological conservatism. Across 45 studies, most relied on unimodal EEG or fNIRS, functional connectivity analyses, and tasks targeting executive and social cognition, while multimodal measurements, effective connectivity, and machine-learning approaches remained rare. Remote digital collaboration was typically operationalized through study designs that isolate or reconfigure interaction channels, highlighting the need for systematic comparisons across graded levels of digitalization. Such comparisons are crucial for investigating how specific technological affordances, altering interaction channels and consequently social cues, shape interpersonal neural, physiological, and behavioral dynamics.

In summary, these patterns highlight both the adaptability of hyperscanning to emerging technological contexts and the limitations that prevent the field from accessing deeper mechanistic explanations of digitally mediated interaction and collaboration. To accelerate progress, we outline reporting practices aimed at improving transparency, reproducibility, and cross-study comparability, and introduce InterBrainDB, a living open-access resource for organizing emerging hyperscanning studies across modalities, paradigms, and analytic approaches.

Future work will benefit from greater multimodal integration, physiologically grounded data fusion strategies, and machine-learning models capable of capturing non-linear and temporally evolving dependencies across brains, bodies, and digital environments. Progress also requires a more fine-grained coverage of cognitive processes to distinguish how each subprocess unfolds within digitally mediated collaboration.

Advancing these methodological and conceptual frontiers is essential for building comprehensive, ecologically grounded models of collaborative behavior in the digital age. Such models will allow cognitive neuroscience to move beyond mapping synchrony toward understanding the mechanisms by which humans coordinate through and with technology.

## Data Availability

Publicly available datasets were analyzed in this study. This data can be found at: https://websites.fraunhofer.de/interbraindb/.
