# Supplementary material for: Reviewing digital collaborative interactions with multimodal hyperscanning through an ever-growing database
Source: Front Neuroergon. 2026 Feb 10;7:1756956. doi: 10.3389/fnrgo.2026.1756956 (PMC12929548; doi:10.3389/fnrgo.2026.1756956)
Supplement: Supplementary file 1 [file Data_Sheet_1.pdf]

Supplementary Material

1 Supplementary Figures and Tables

Supplementary Table 1
Exact Search Terms for Each Database

Engine	Number of results	Search term
Scopus	5,502	(hyperscan* OR "social neuroscience" OR "two-person neuroscience" OR interbrain OR interpersonal OR "brain-to-brain interaction" OR interneural OR inter-subject OR synchron* OR coupling OR "functional connectivity" OR "effective connectivity") AND (flow OR team OR "team flow" OR "work flow" OR "team performance" OR "team engagement" OR "group flow" OR "collective flow" OR "team dynamics" OR "group performance" OR "group dynamics" OR "collaborative engagement" OR "collaborative performance" OR "collective efficacy") AND (fnirs OR "functional near-infrared spectroscopy" OR eeg OR electroencephalogra* OR ecg OR electrocardiogra* OR ppg OR photoplethysmogra* OR eda OR "electrodermal activity" OR "heart rate" OR pulse OR "skin conductance" OR eye-track* OR "eye tracking" OR "gaze tracking" OR physiolog* OR multimodal* OR "multi-modal") AND (remote OR virtual OR online OR web-based) AND (LIMIT-TO (SRCTYPE , "j")) AND (LIMIT-TO (DOCTYPE , "ar") OR EXCLUDE (DOCTYPE , "re") OR EXCLUDE (DOCTYPE , "tb") OR EXCLUDE (DOCTYPE , "cr")) AND (LIMIT-TO (SUBJAREA , "NEUR")) AND (LIMIT-TO (LANGUAGE , "English")) AND (EXCLUDE (EXACTKEYWORD , "Alzheimer Disease") OR EXCLUDE (EXACTKEYWORD , "Animal") OR EXCLUDE (EXACTKEYWORD , "Animal Cell") OR EXCLUDE (EXACTKEYWORD , "Animal Experiment") OR EXCLUDE (EXACTKEYWORD , "Animal Model") OR EXCLUDE (EXACTKEYWORD , "Animal Tissue") OR EXCLUDE (EXACTKEYWORD , "Animals") OR EXCLUDE (EXACTKEYWORD , "Macaque") OR EXCLUDE (EXACTKEYWORD , "Monkey") OR EXCLUDE (EXACTKEYWORD , "Child") OR EXCLUDE (EXACTKEYWORD , "Rat") OR EXCLUDE (EXACTKEYWORD , "Rats") OR EXCLUDE (EXACTKEYWORD , "Stroke") OR EXCLUDE (EXACTKEYWORD , "Parkinson Disease") OR EXCLUDE (EXACTKEYWORD , "Depression") OR EXCLUDE (EXACTKEYWORD , "Schizophrenia") OR EXCLUDE (EXACTKEYWORD , "Nerve Cell Plasticity") OR EXCLUDE (EXACTKEYWORD , "Pathology") OR EXCLUDE (EXACTKEYWORD , "Epilepsy") OR EXCLUDE (EXACTKEYWORD , "Cancer") OR EXCLUDE (EXACTKEYWORD , "Stem Cells"))
Pubmed	441	(hyperscan* OR "social neuroscience" OR "two-person neuroscience" OR interbrain OR interpersonal OR "brain-to-brain interaction" OR interneural OR inter-subject OR synchron* OR coupling OR "functional connectivity" OR "effective connectivity") AND (flow OR team OR "team flow" OR "work flow" OR "team performance" OR "team engagement" OR "group flow" OR "collective flow" OR "team dynamics" OR "group performance" OR "group dynamics" OR "collaborative engagement" OR "collaborative performance"

		OR "collective efficacy") AND (fnirs OR "functional near-infrared spectroscopy" OR eeg OR electroencephalogra* OR ecg OR electrocardiogra* OR ppg OR photoplethysmogra* OR eda OR "electrodermal activity" OR "heart rate" OR pulse OR "skin conductance" OR eye-track* OR "eye tracking" OR "gaze tracking" OR physiolog* OR multimodal* OR "multi-modal") AND (remote OR virtual OR online OR web-based)
Web of Science	332	TS=(hyperscan* OR "social neuroscience" OR "two-person neuroscience" OR interbrain OR interpersonal OR "brain-to-brain interaction" OR interneuron OR inter-subject OR synchron* OR coupling OR "functional connectivity" OR "effective connectivity") AND TS=(flow OR team OR "team flow" OR "work flow" OR "team performance" OR "team engagement" OR "group flow" OR "collective flow" OR "team dynamics" OR "group performance" OR "group dynamics" OR "collaborative engagement" OR "collaborative performance" OR "collective efficacy") AND TS=(fnirs OR "functional near-infrared spectroscopy" OR eeg OR electroencephalogra* OR ecg OR electrocardiogra* OR ppg OR photoplethysmogra* OR eda OR "electrodermal activity" OR "heart rate" OR pulse OR "skin conductance" OR eye-track* OR "eye tracking" OR "gaze tracking" OR physiolog* OR multimodal* OR "multi-modal") AND TS=(remote OR virtual OR online OR web-based)

Supplementary Table 2
Interaction Conditions Across Modalities

Modality	Total conditions	Unique IM-IS combination s	Cross-condition occurrences	Constant IM, varying IS	Constant IS, varying IM	Both varying	Digital vs non-digital comparison	With verbal IM	Without verbal IM
All	75	22	16	6	1	9	8	19	26
FNIRS	20	10	6	2	0	4	4	6	6
EEG	33	14	6	2	1	3	3	5	14
Eye-tracking	15	7	3	2	0	1	0	6	3

Note. The distribution of interaction conditions regarding interaction scenarios (IS) and interaction medium (IM) displayed for all included studies as well as for most used measurement modalities fNIRS, EEG, and eye-tracking. From left to right, the Table displays the total number of conditions, the number of unique IM and IS combinations, the total counted cross-condition occurrences (i.e., whether the same study compared multiple conditions), the number of cross-condition occurrences with either IM or IS held constant, the number of condition with both interaction dimensions varying, the number of cross-condition occurrences where digital and non-digital interaction dimensions were compared, and finally the number of studies with and without verbal IM.

Supplementary Figure 1

Distribution of Hyperscanning Conditions Across Interaction Types and Combined Modalities

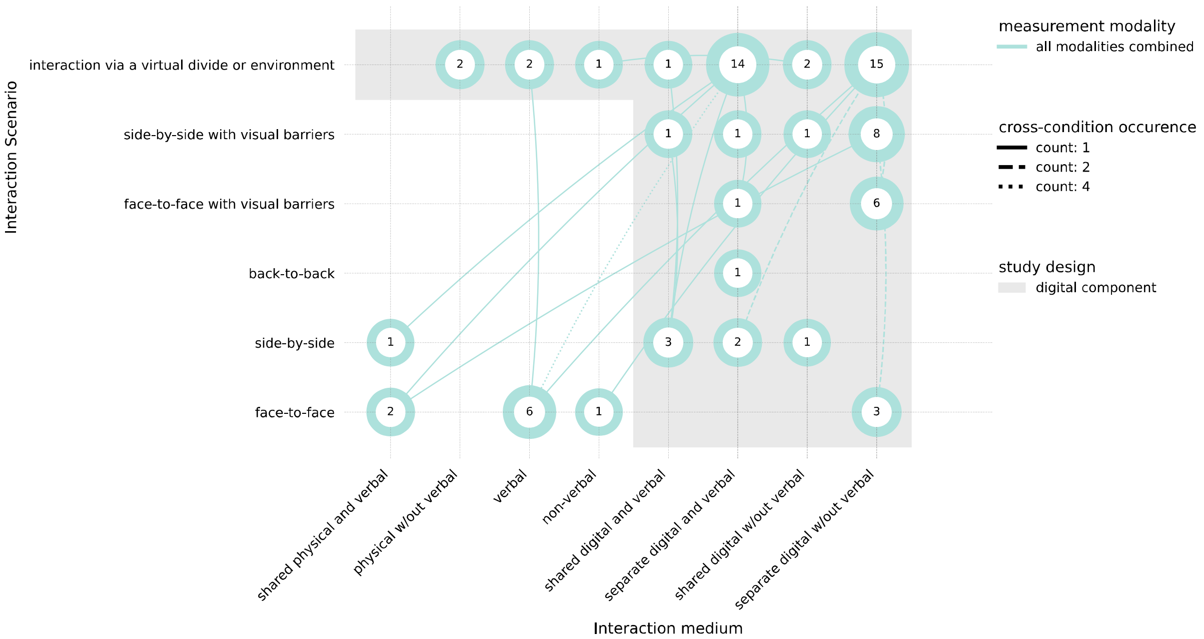

Note. The cross-sectional distribution of all hyperscanning conditions of 45 studies across interaction medium and interaction scenario axes. The numbers in circles provide the counted occurrences (n = 75 conditions) of the cross-section of an interaction medium and scenario (n = 22 unique combinations, shown as circles). The connection lines indicate reported cross-condition occurrences separated by axis (n = 16 simultaneous condition occurrences). Studies involving a digital component either through a digital manipulation or virtual interaction scenario are marked through a gray shaded area. Note that conditions that do not fall within this gray area are part of a comparison to a condition with a digital component.

Supplementary Figure 2

Distribution of FNIRS Hyperscanning Conditions Across Interaction Types

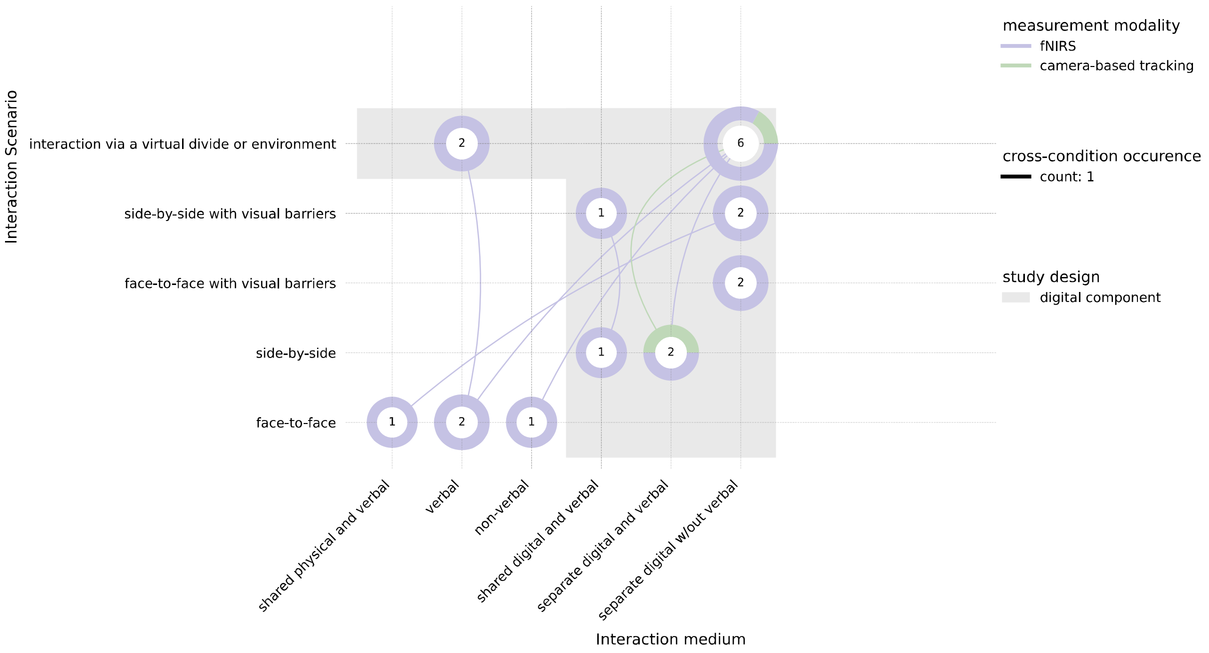

Note. The cross-sectional distribution of all hyperscanning conditions of 12 studies across interaction medium and interaction scenario axes. The numbers in circles provide the counted occurrences (n = 20 conditions across modalities) of the cross-section of an interaction medium and scenario (n = 10 unique combinations, shown as circles). The colors represent the measurement modalities reported for each cross-section of conditions. The connection lines indicate reported cross-condition occurrences separated per axis (n = 6 simultaneous condition occurrences). Studies involving a digital component either through a digital medium or virtual interaction scenario are marked through a gray shaded area. There were eight instances of condition co-occurrence within the same study, two with constant interaction medium and varying interaction scenario (Yamaya et al., 2025; Huan Zhang et al., 2023), none with constant interaction scenario and varying interaction medium, and three where both interaction medium and interaction scenario were varied (J. Liu et al., 2019; Lu et al., 2020; Shih et al., 2024). Furthermore, one study introduced face-to-face, side-by-side with visual barriers, and completely virtually remote scenarios and had participants interact with physical objects and with verbal communication in the face-to-face condition and separate digital interactions without verbal communication in the other two (J. Wu et al., 2025). Five instances of directly comparing digital and non-digital conditions were found (J. Liu et al., 2019; Lu et al., 2020; J. Wu et al., 2025; Yamaya et al., 2025). Six studies included verbal communication (Balters et al., 2023; Lu et al., 2020; Shih et al., 2024; J. Wu et al., 2025; Yamaya et al., 2025; Huan Zhang et al., 2023) and nine studies involved non-verbal communication (X. Cheng et al., 2019; Hayne et al., 2023; Hu et al., 2017; J. Liu et al., 2019; Lu et al., 2020; Pan et al., 2017; Shih et al., 2024; J. Wu et al., 2025; Hejing Zhang et al., 2023).

Supplementary Figure 3

Distribution of EEG Hyperscanning Conditions Across Interaction Types

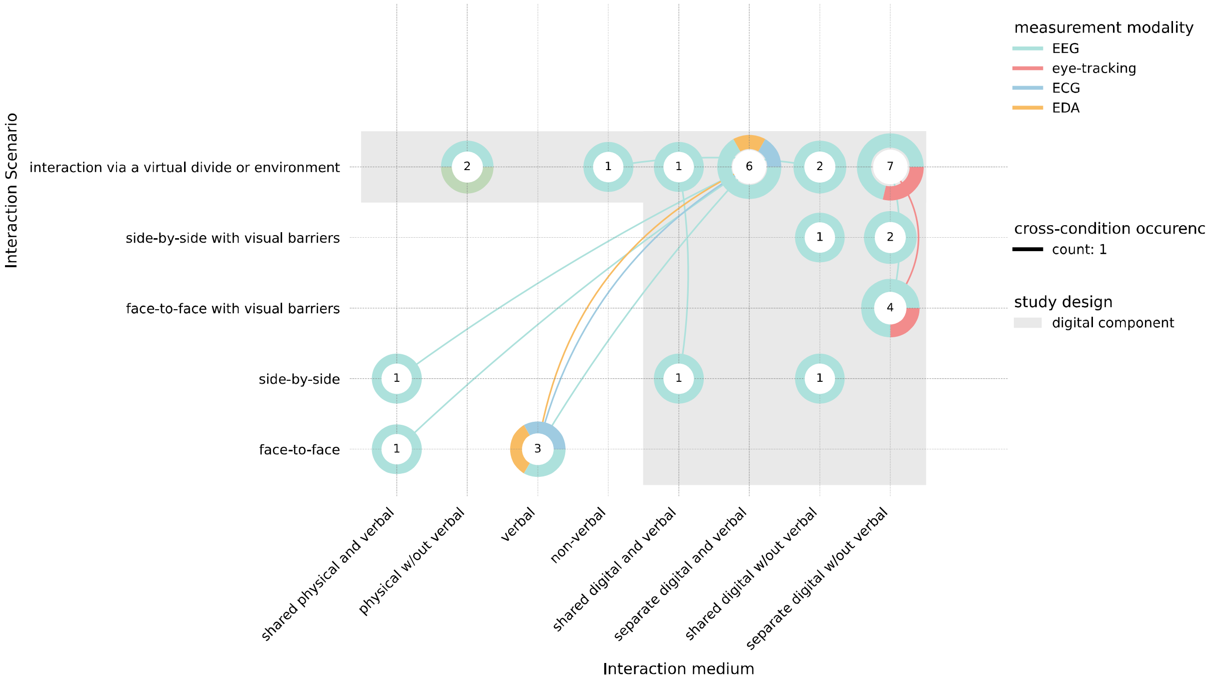

Note. The cross-sectional distribution of studies including EEG hyperscanning conditions of 19 studies across interaction medium and interaction scenario axes. The numbers in circles provide the counted occurrences (n = 33 conditions across modalities) of the cross-section of an interaction medium and scenario (n = 14 unique combinations, shown as circles). The colors represent the measurement modalities reported for each cross-section of conditions. The connection lines indicate reported cross-condition occurrences separated per axis (n = 6 simultaneous condition occurrences). Studies involving a digital component either through a digital medium or virtual interaction scenario are marked through a gray shaded area. Note that conditions that do not fall within this gray area are part of a comparison to a condition with a digital component. Six studies involved co-occurrences of such unique combinations within the same experiment, two with constant interaction medium and varying interaction scenario (C.-H. Chuang & Hsu, 2023; Hayati et al., 2025), one with constant interaction scenario and varying interaction medium (Gumilar et al., 2021), and three where both varied (Balconi et al., 2023; Balconi et al., 2022; Cross et al., 2022). The latter three co-occurrences involved digital versus non-digital components. Most EEG studies included non-verbal communication (n = 14; Antonenko et al., 2019; Astolfi et al., 2020; Chen et al., 2020; C.-H. Chuang & Hsu, 2023; T.-M. Chuang et al., 2024; Ciaramidaro et al., 2024; Flösch et al., 2024; Gugnowska et al., 2022; Gumilar et al., 2021;Léné et al., 2021; Y. Liu et al., 2024; Z. Wang et al., 2024; Wikström, 2022; D. Zhang et al., 2019; Zhou et al., 2021), although five studies reported findings involving verbal communication (Balconi et al., 2023; Balconi et al., 2022; Cross et al., 2022; Du et al., 2022; Hayati et al., 2025).

Supplementary Figure 4

Distribution of Eye-Tracking Hyperscanning Conditions Across Interaction Types

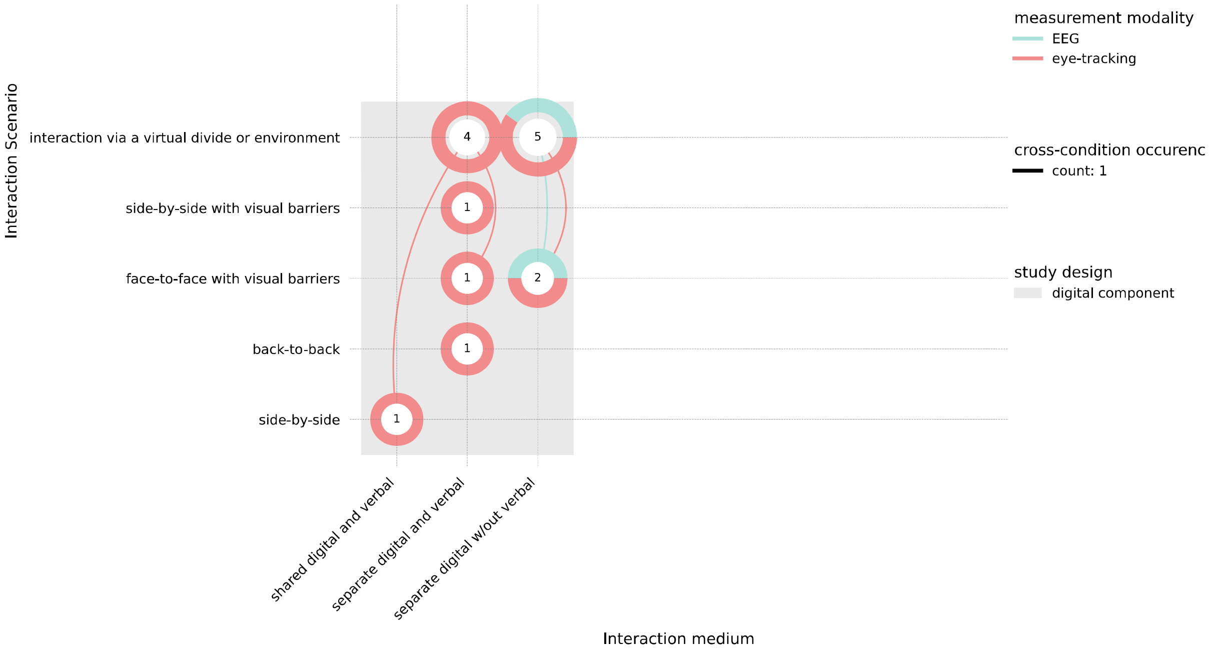

Note. The cross-sectional distribution of studies including eye-tracking hyperscanning conditions of 9 studies across interaction medium and interaction scenario axes. The numbers in circles provide the counted occurrences (n = 15 conditions across modalities) of the cross-section of an interaction medium and scenario (n = 7 unique combinations, shown as circles). The colors represent the measurement modalities reported for each cross-section of conditions. The connection lines indicate reported cross-condition occurrences separated per axis (n = 3 simultaneous condition occurrences for eye-tracking). Studies involving a digital component either through a digital medium or virtual interaction scenario are marked through a gray shaded area. Eye-tracking studies demonstrated fewer comparative connections between interaction modes. 15 conditions with six unique combinations of interaction medium and scenario were found. Two of these studies included varying scenarios with constant manipulation (C.-H. Chuang & Hsu, 2023; Špakov et al., 2019), one study varied both (Wisiecka et al., 2023). Of the nine eye-tracking studies identified, three focused on non-verbal interactions (C.-H. Chuang & Hsu, 2023; Fındık-Coşkunçay & Çakır, 2022; Z. Wang et al., 2024), while six included verbal communication (S. Cheng et al., 2022; Hoffmann et al., 2024; Kütt et al., 2019; Pöysä-Tarhonen et al., 2021; Špakov et al., 2019; Wisiecka et al., 2023). Importantly, all eye-tracking studies incorporated some form of digital manipulation, with participants either visually separated or interacting in a virtual context. There were no studies in which both digital and analog conditions (whether interaction scenario or interaction medium) were present within the same experiment.

Supplementary Figure 5

Category Counts of Analysis Methods and Cognitive Functions

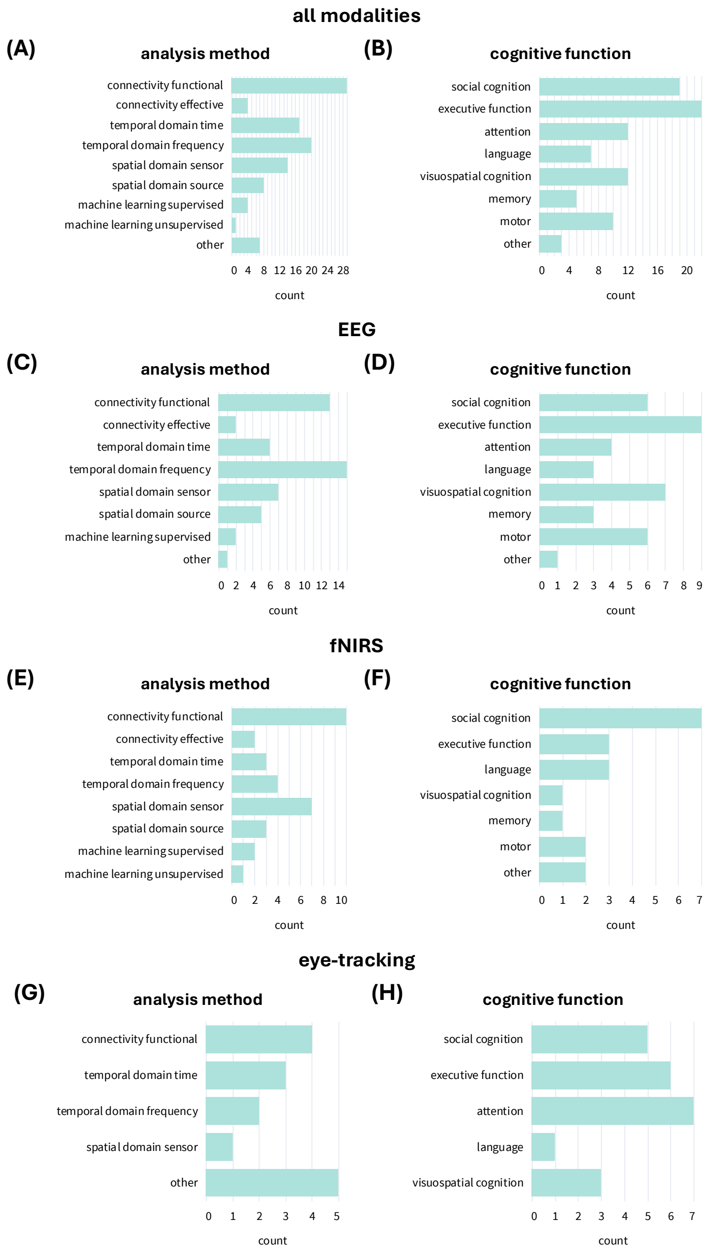

Note. (A) Analysis methods were distributed across eight categories. The category ‘other’ included two studies assessing the amount of gaze-sharing, two studies analyzing gaze patterns, and one study measuring muscle co-contraction (B) Cognitive functions were distributed across eight categories. The category ‘other’ included two studies focusing on joint designing ability and one study assessing joint musical ability. (C-D) Analysis methods and cognitive functions counted in EEG studies. (E-F) Analysis methods and cognitive functions counted in fNIRS studies. (G-H) Analysis methods and cognitive functions counted in eye-tracking studies.
